# Supplementary material for: Replication‐induced DNA secondary structures drive fork uncoupling and breakage
Source: EMBO J. 2023 Oct 2;42(22):e114334. doi: 10.15252/embj.2023114334 (PMC10646557; doi:10.15252/embj.2023114334)
Supplement: Supplementary file 1 — Appendix [file EMBJ-42-e114334-s001.pdf]

TABLE OF CONTENTS

1. Appendix Figure S1...2
2. Appendix Figure S2...3
3. Appendix Figure S3...4
4. Appendix Figure S4...5
5. Appendix Figure S5 (A-D)..6
6. Appendix Figure S5 (E-J)...7

Appendix Figure S1

A

G4 mutated substrates	Sequence (5' - 3')
c-MYC	TGAGGGTGGGTAGGGTGGGTAA
c-MYC MUT	TGAGAGTGAGTAGAGTGAGTAA
(GGGT)$_4$	GGGTGGGTGGGTGGGT
(GGGT)$_3$	GGGTGGGTGGGT
CEB25 L111(T) (Piazza et al., 2015)	AAGGGTGGGTGGGTGGGTGTGAGT GTGGGTGTGGAGGTAGATGT
CEB25 L111(A) (Piazza et al., 2015)	AAGGGAGGGAGGGAGGGTGTGAG TGTGGGTGTGGAGGTAGATGT

B

iMotif mutated substrates	Sequence (5'-3')
DUX4L22	CCCCCGAAACGCGCCCCCCTCCCCCCTC CCCCCTCTCCCCC
DUX4L22 MUT	CCTCCGAAACGCGCCTTCCTCCTTCCT CCTTCCTCTCCTCC
SNORD112	CCCCCCCCCGCCCCCCACCCCCCCACCCC CCCCCCC
SNORD112 MUT	CCTCCTTCCGCCTTCCACCTTCTCACC TCCTCCTCC

E

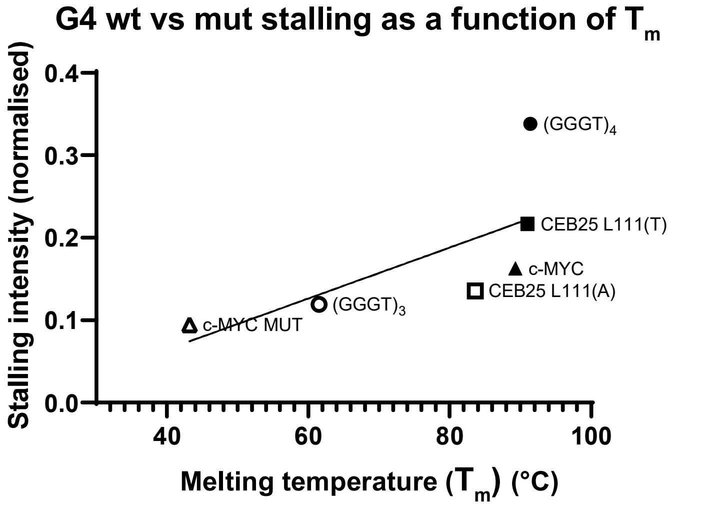

F

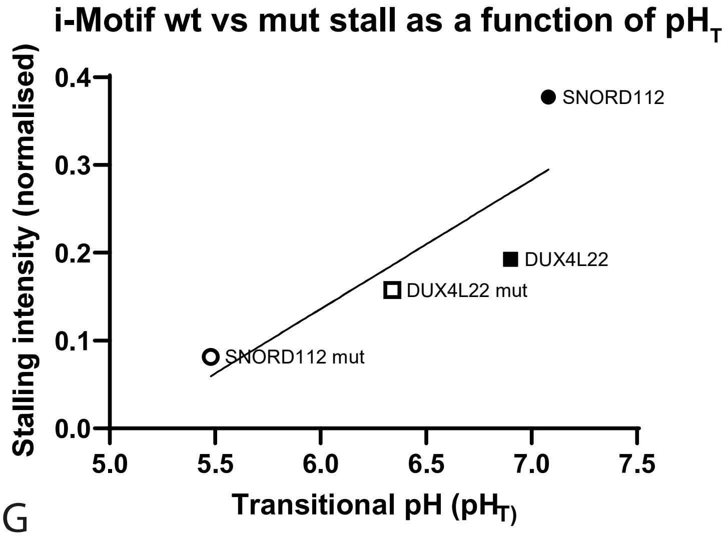

C

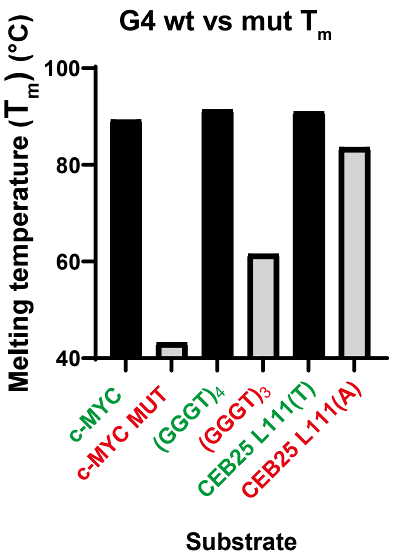

D

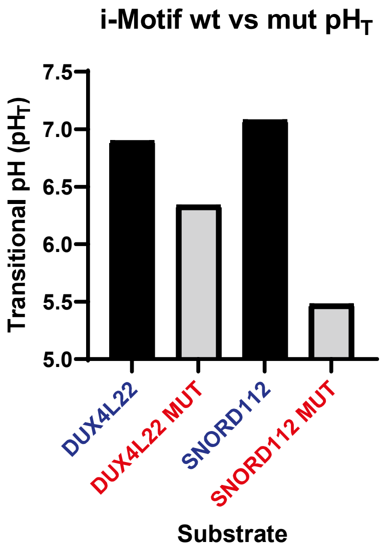

G

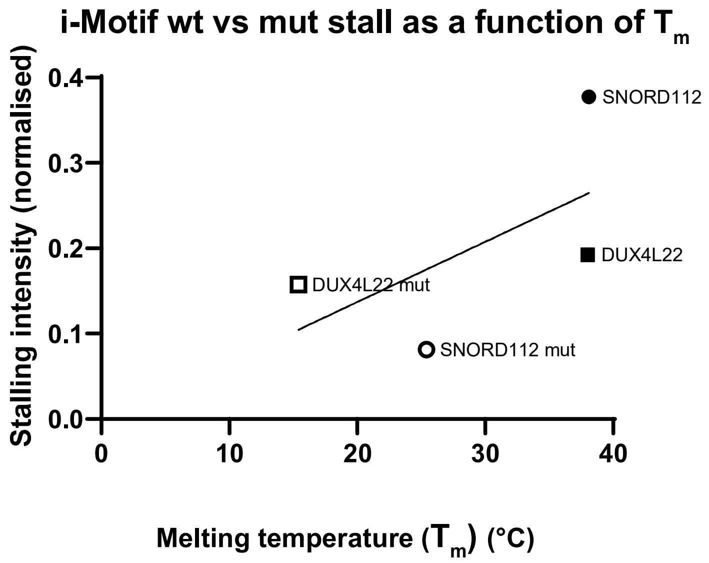

Appendix Figure S1. Correlations between biophysical properties of mutated G4 and iMs and replisome stalling. (A and B) Sequences of wildtype or mutated G4 (A) or iM-forming (B) sequences used to generate substrates for replication reactions in Fig. 1D and 1F. Mutated sequences (depicted in red) have abrogated or weakened ability to form structures. (C) Melting temperatures of the secondary structures formed by sequences depicted in Fig. 1D. (D) The transitional pH (pHT) of the structures formed by the sequences depicted in Fig. 1F. (E) Melting temperatures of the structures formed by sequences tested in Fig. 1D versus their stalling intensities as calculated in Fig. 1E. Line indicates simple linear regression with Pearson correlation r value of 0.7. (F and G) Transitional pH (pH_T) (F) or melting temperature (G) of the structures formed by sequences tested in Fig. 1F versus their stalling intensities as calculated in Fig. 1G. Lines indicate simple linear regression with Pearson correlation r values of 0.8 (F) and 0.6 (G).

Appendix Figure S2

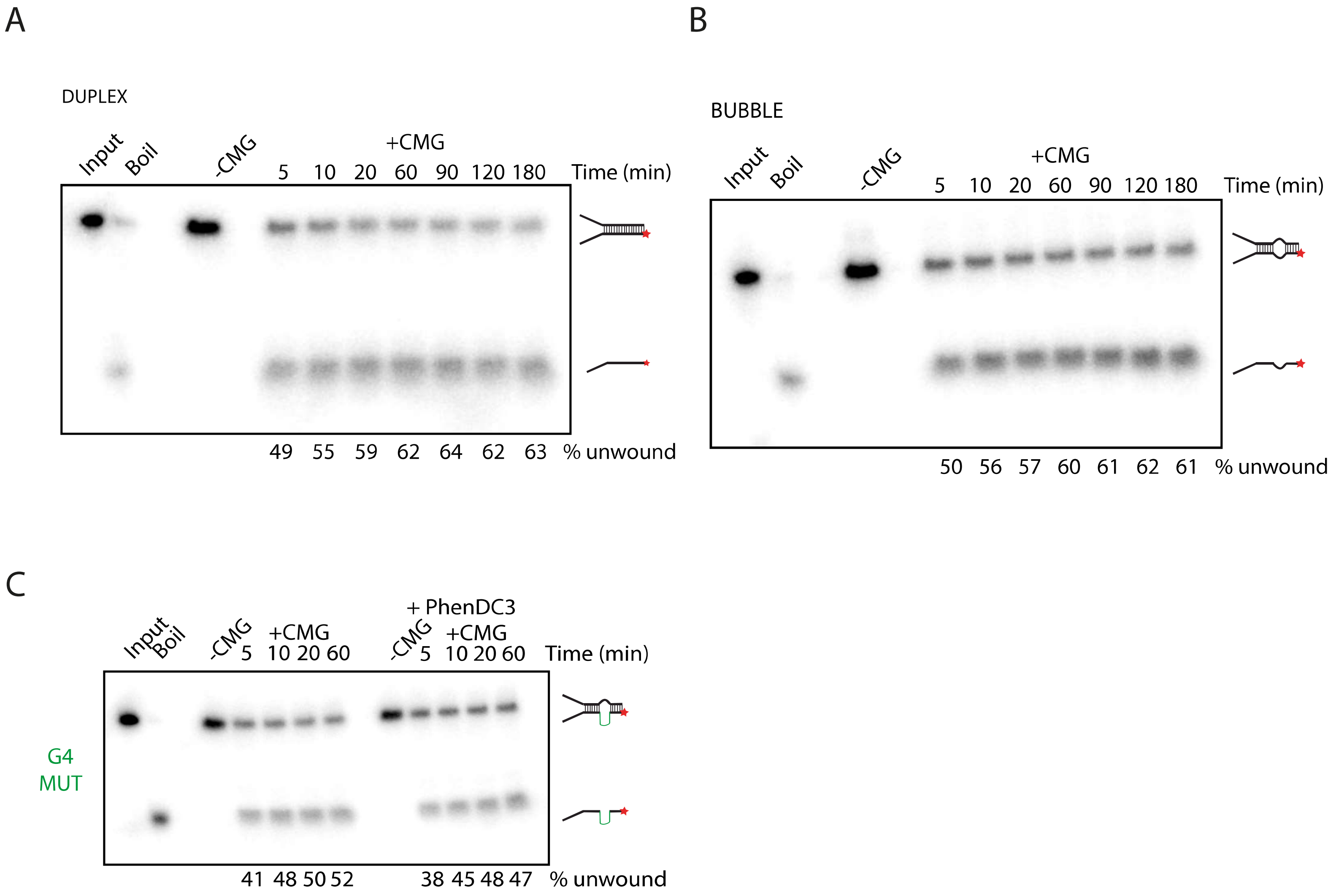

Appendix Figure S2. Unwinding of non-structure containing substrates by CMG.
(A-B) CMG unwinding assays on substrates containing duplex (A) or a poly(dT)$_{19}$ bubble (B). CMG unwinding was stimulated by the addition of 2 nM ATP following CMG loading in the presence of ATPγS. Samples were taken at the indicated time points. Products were run on 10% TBE gels. Input and boiled substrates were used as controls to visualise where original and unwound substrates run on the gel. The proportion of template unwound was calculated by measuring the intensity of the 'unwound' product band as a proportion of the total product intensity for each lane. (C) CMG unwinding assays on substrates containing G4 mutant sequence. Reactions were carried out as in (B) but with the addition of 0.25 μM PhenDC3 where indicated.

Appendix Figure S3

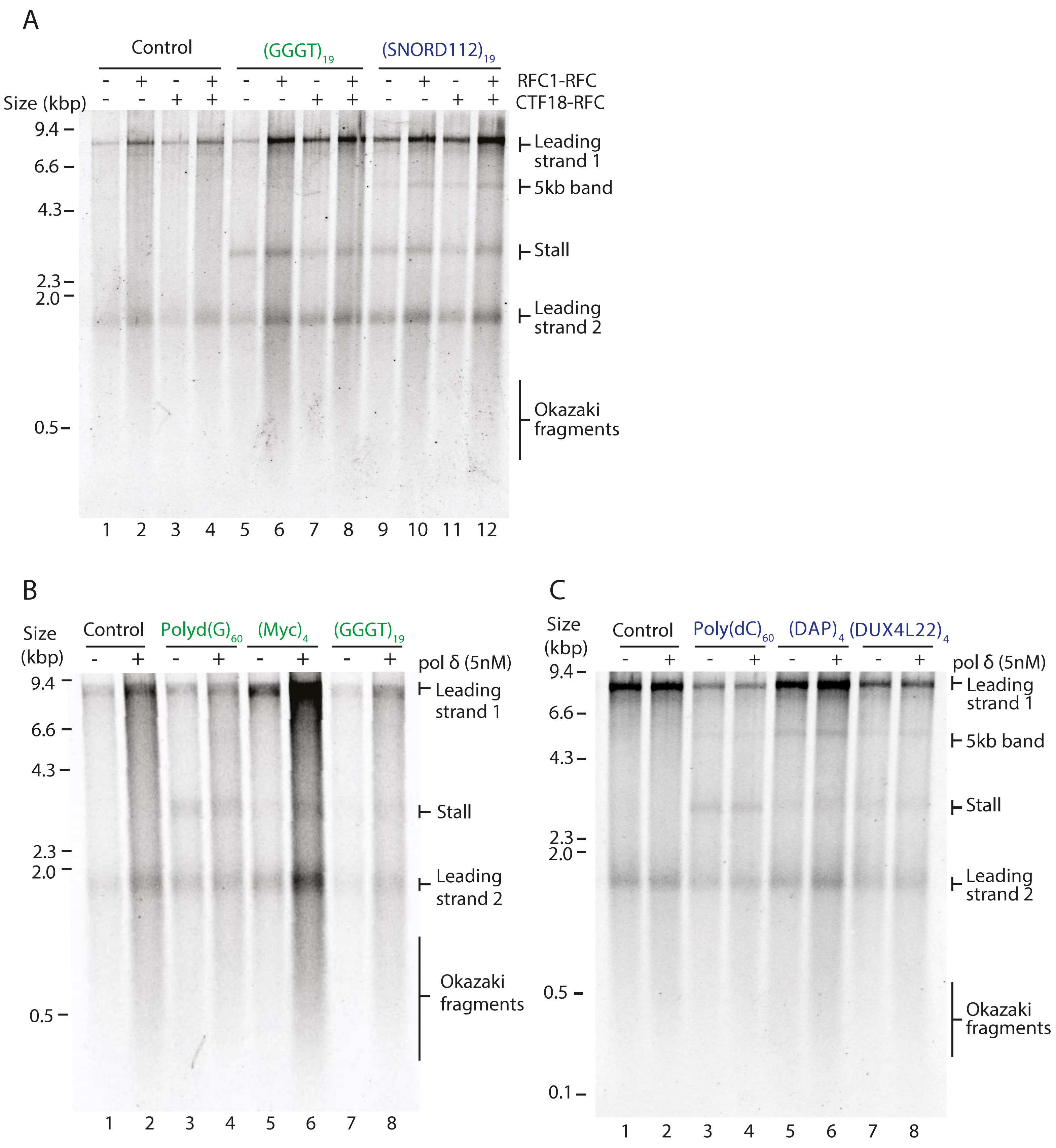

Appendix Figure S3. Pol δ and CTF18-RFC do not affect replisome stalling at G4s or iMs.
(A) Replication of substrates containing a G4 ((GGGT)$_{19}$) or iM ((SNORD112)$_1$) in the presence or absence of RFC1-RFC or CTF18-RFC as indicated. Products were visualised on a denaturing agarose gel.
(B and C) Analysis of replication products of G4 (B) or iM (C) substrates in the presence or absence of pol δ on denaturing agarose gels.

Appendix Figure S4

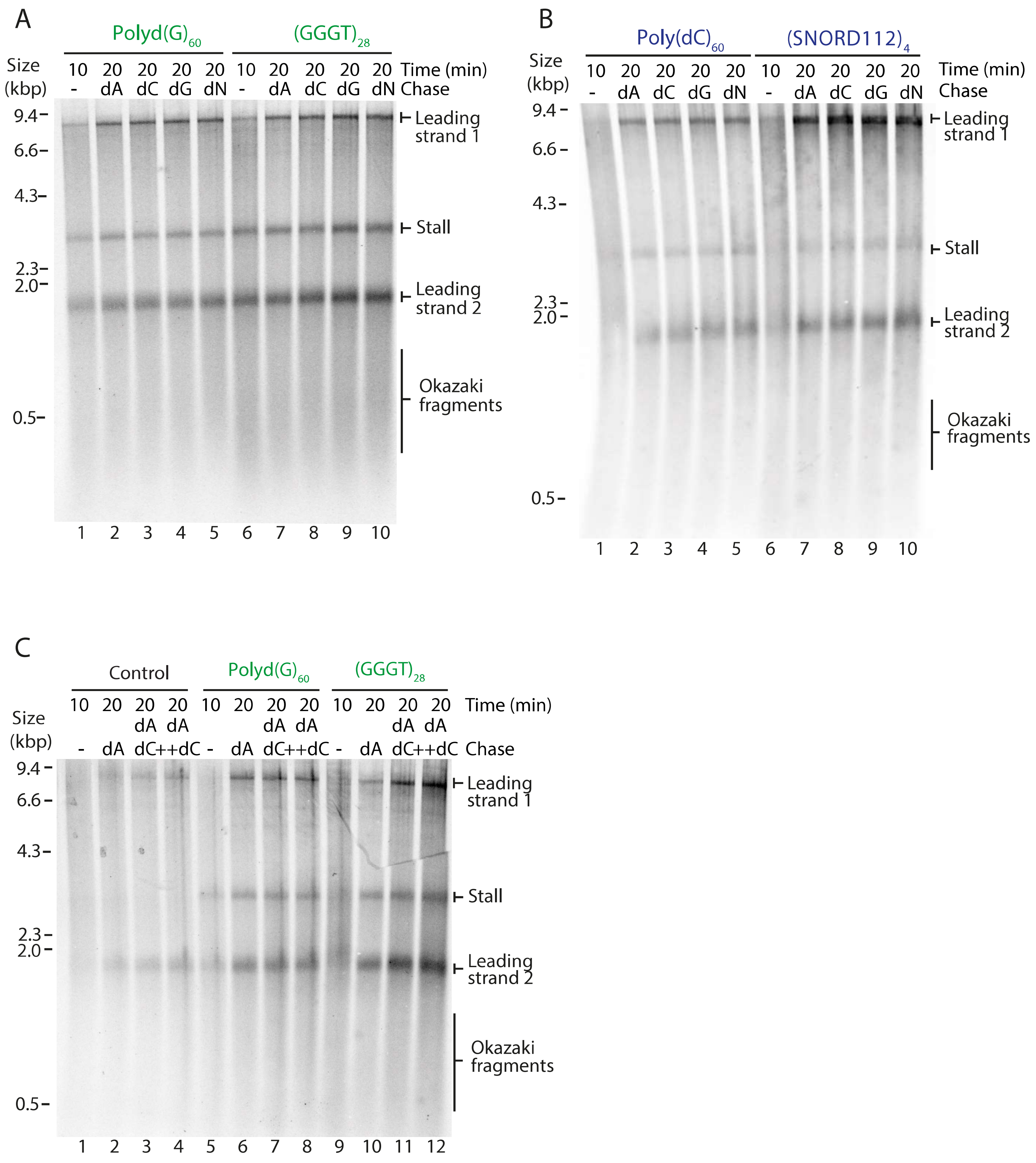

Appendix Figure S4. Excess dNTPs can not rescue stalling at G4s or iMs.
(A and B) Pulse-chase experiments carried out with the indicated templates.
Reactions were initiated with radiolabelled dATP for 10 min and chased for another 10 min with either excess 'cold' dATP alone (dA) or in combination with dCTP (dC), dGTP (dG), or all four dNTPs (dN). (C) As per A and B but reactions were chased with either 'cold' dATP alone or in combination with the same excess of dCTP or with double the excess of dCTP as dATP.

Appendix Figure S5

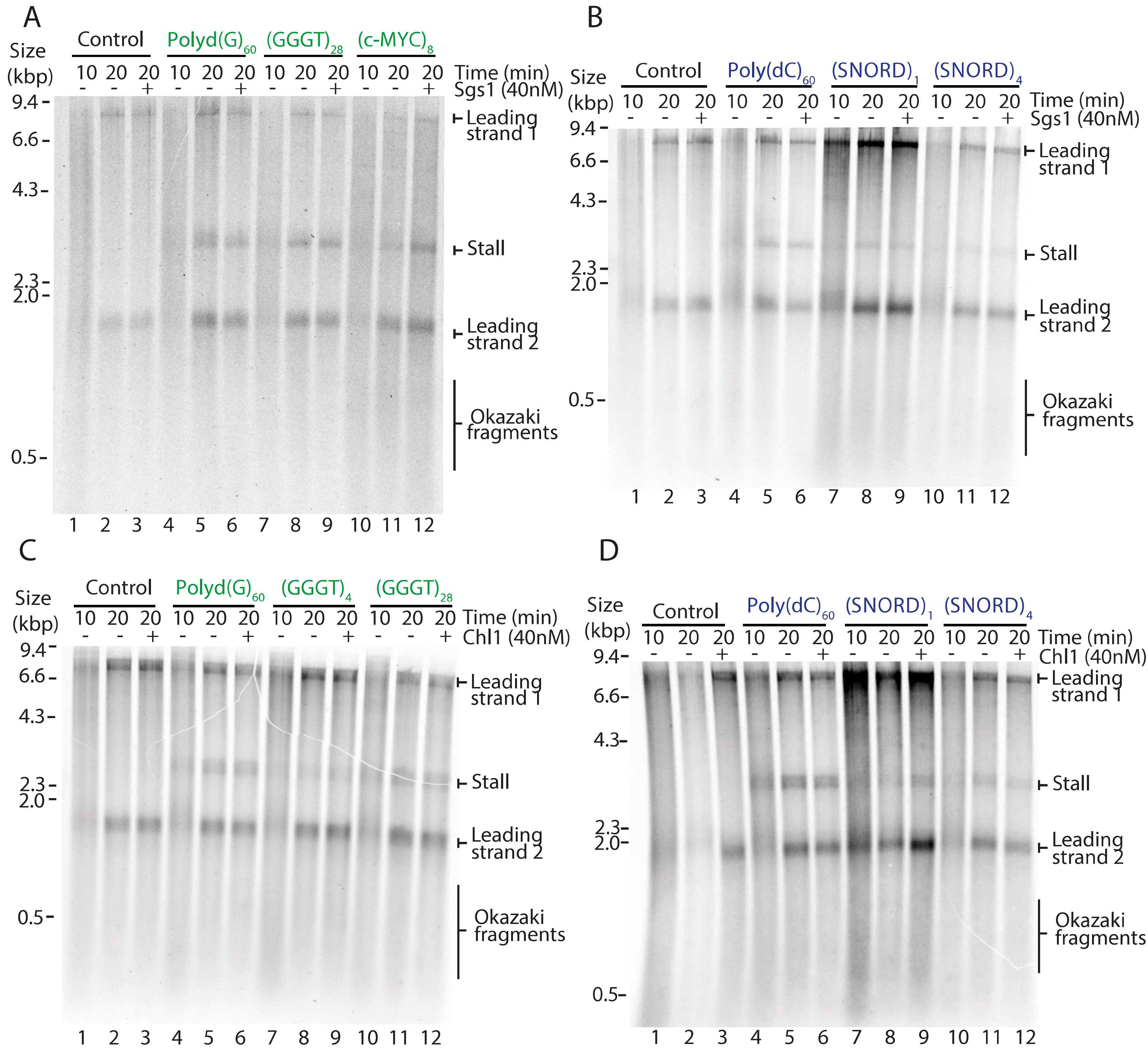

Appendix Figure S5. Sgs1, Chl1, Rrm3 and Hrq1 are unable to rescue replication stalling at G4s or iMs. (A-F) Pulse-chase experiments carried out with the indicated templates. Reactions were initiated with radiolabelled dATP. After a 10 min pulse, either Sgs1 (A and B), Chl1 (C and D), Rrm3 (E) or Hrq1 (F) (wildtype or an ATPase dead mutant, K318A) was added with the chase and samples taken after another 10 min. (G-J) Helicase unwinding assays using purified Sgs1 (G), Chl1 (H), Rrm3 (I) or Hrq1 (wildtype or K318A mutant) (J) . Time points were taken as indicated. Products were run on 10% TBE gels. Input and boiled substrates were used as controls to visualise where original and unwound substrates run on the gel. Assays demonstrate that both Sgs1 and Chl1 show robust unwinding activity, Rrm3 and Hrq1 show partial activity.

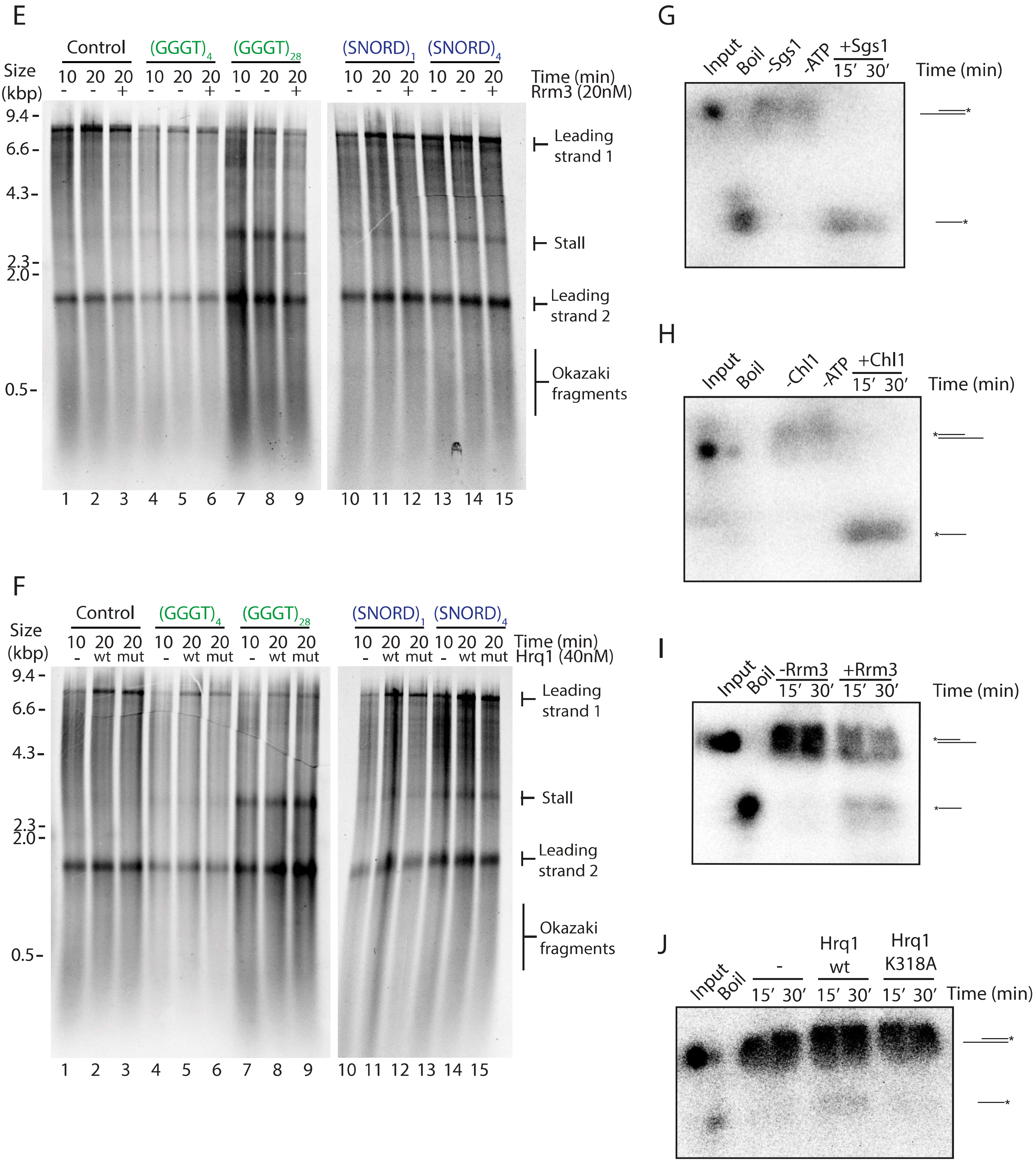
E
Control
(GGGT)4
(GGGT)28
(SNORD)1
(SNORD)4
Size (kbp)
10 20 20 10 20 20 10 20 20 10 20 20 10 20 20 Time (min)
- - + - - + - - + - - + - - + Rrm3 (20nM)
9.4
6.6
4.3
2.3
2.0
0.5
Leading strand 1
Stall
Leading strand 2
Okazaki fragments
1 2 3 4 5 6 7 8 9 10 11 12 13 14 15
F
Control
(GGGT)4
(GGGT)28
(SNORD)1
(SNORD)4
Size (kbp)
10 20 20 10 20 20 10 20 20 10 20 20 10 20 20 Time (min)
- wt mut - wt mut - wt mut - wt mut - wt mut Hrq1 (40nM)
9.4
6.6
4.3
2.3
2.0
0.5
Leading strand 1
Stall
Leading strand 2
Okazaki fragments
1 2 3 4 5 6 7 8 9 10 11 12 13 14 15
G
Input
Boil
-Sgs1
-ATP
+Sgs1
15′ 30′
Time (min)
H
Input
Boil
-Chl1
-ATP
+Chl1
15′ 30′
Time (min)
I
Input
Boil
-Rrm3
15′ 30′
+Rrm3
15′ 30′
Time (min)
J
Input
Boil
-
15′ 30′
Hrq1 wt
15′ 30′
Hrq1 K318A
15′ 30′
Time (min)
