# Supplementary material for: Replication‐induced DNA secondary structures drive fork uncoupling and breakage
Source: EMBO J. 2023 Oct 2;42(22):e114334. doi: 10.15252/embj.2023114334 (PMC10646557; doi:10.15252/embj.2023114334)
Supplement: Supplementary file 2 — Expanded View Figures PDF [file EMBJ-42-e114334-s005.pdf]

Expanded View Figures

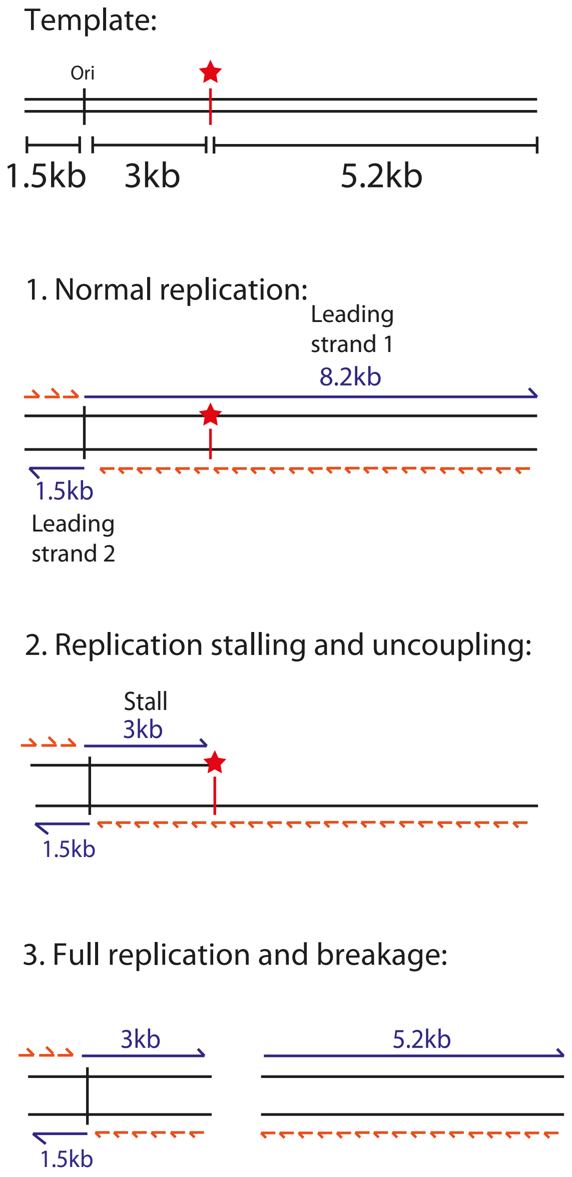

Figure EV1. Schematic of potential replication products from G4 and iM-containing templates.

Position of the origin of replication is marked as 'ori' from which replication initiates through sequence-specific replisome loading. Two forks initiate from the origin and generate one longer rightward moving fork of 8.2 kb ('Leading strand 1') and one shorter leftward moving fork of 1.5 kb ('Leading strand 2'). Lagging strand products are synthesised as short Okazaki fragments. The multiple cloning site (indicated by a red star) is positioned 3 kb downstream of the origin and was used to insert various G4 or iM-forming sequences into the template. This means that only the rightward moving fork would encounter them and therefore 'leading strand 2' can serve as an internal control. Under conditions of replication stalling, helicase-polymerase uncoupling may occur which may be associated with intrinsic repriming at the site of fork stalling. Replication products may also break at the site of the insert during or after replication.

© 2023 The Authors

Figure EV2. Quantification of replisome stalling at G4s and iMs in relation to their biophysical characteristics.

A Quantification of stall intensities from three independent experiments of substrates, as shown in Fig 1B. The 3 kb stalling intensity band was normalised to the intensity of 'leading strand 2' in each lane to account for variation in the efficiencies of reactions for each substrate. The mean stalling intensity is plotted with error bars representing the standard error.

B Melting temperatures of the G4s formed by sequences tested in Fig 1B versus their stalling intensities as calculated in (A). Line indicates simple linear regression with a Pearson correlation *r* value of 0.76.

C Quantification of stall intensities as in (A) from three independent experiments of substrates as shown in Fig 1C.

D Melting temperatures of the iMs formed by sequences tested in Fig 1C versus their stalling intensities as calculated in Fig EV2C. Line indicates simple linear regression with Pearson correlation *r* value of −0.34.

E The transitional pH (pH_T) of the iMs formed by the sequences tested in Fig 1C versus their stalling intensities as presented in calculated in Fig EV2C. Line indicates simple linear regression with a Pearson correlation *r* value of 0.27.

 © 2023 The Authors

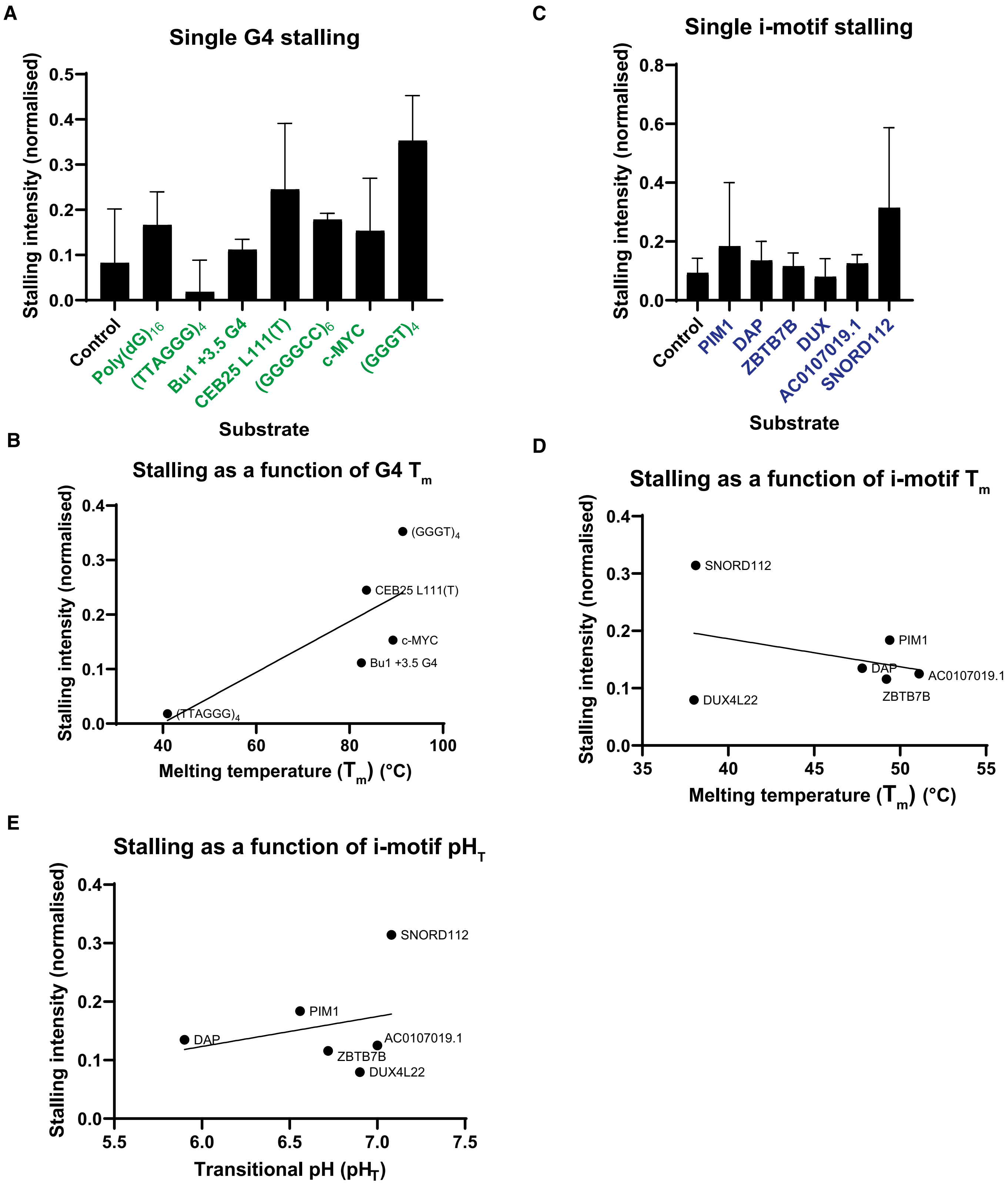
A
Single G4 stalling
Stalling intensity (normalised)
0.5
0.4
0.3
0.2
0.1
0.0
Control
Poly(dG)16
(TTAGGG)4
Bu1 +3.5 G4
CEB25 L111(T)
(GGGGCC)6
c-MYC
(GGGT)4
Substrate
B
Stalling as a function of G4 Tm
Stalling intensity (normalised)
(GGGT)4
CEB25 L111(T)
c-MYC
Bu1 +3.5 G4
(TTAGGG)4
Melting temperature (Tm) (°C)
C
Single i-motif stalling
Stalling intensity (normalised)
Control
PIM1
DAP
ZBTB7B
DUX
AC0107019.1
SNORD112
Substrate
D
Stalling as a function of i-motif Tm
Stalling intensity (normalised)
SNORD112
PIM1
DAP
AC0107019.1
ZBTB7B
DUX4L22
Melting temperature (Tm) (°C)
E
Stalling as a function of i-motif pHT
Stalling intensity (normalised)
SNORD112
PIM1
DAP
AC0107019.1
ZBTB7B
DUX4L22
Transitional pH (pHT)

Figure EV2.

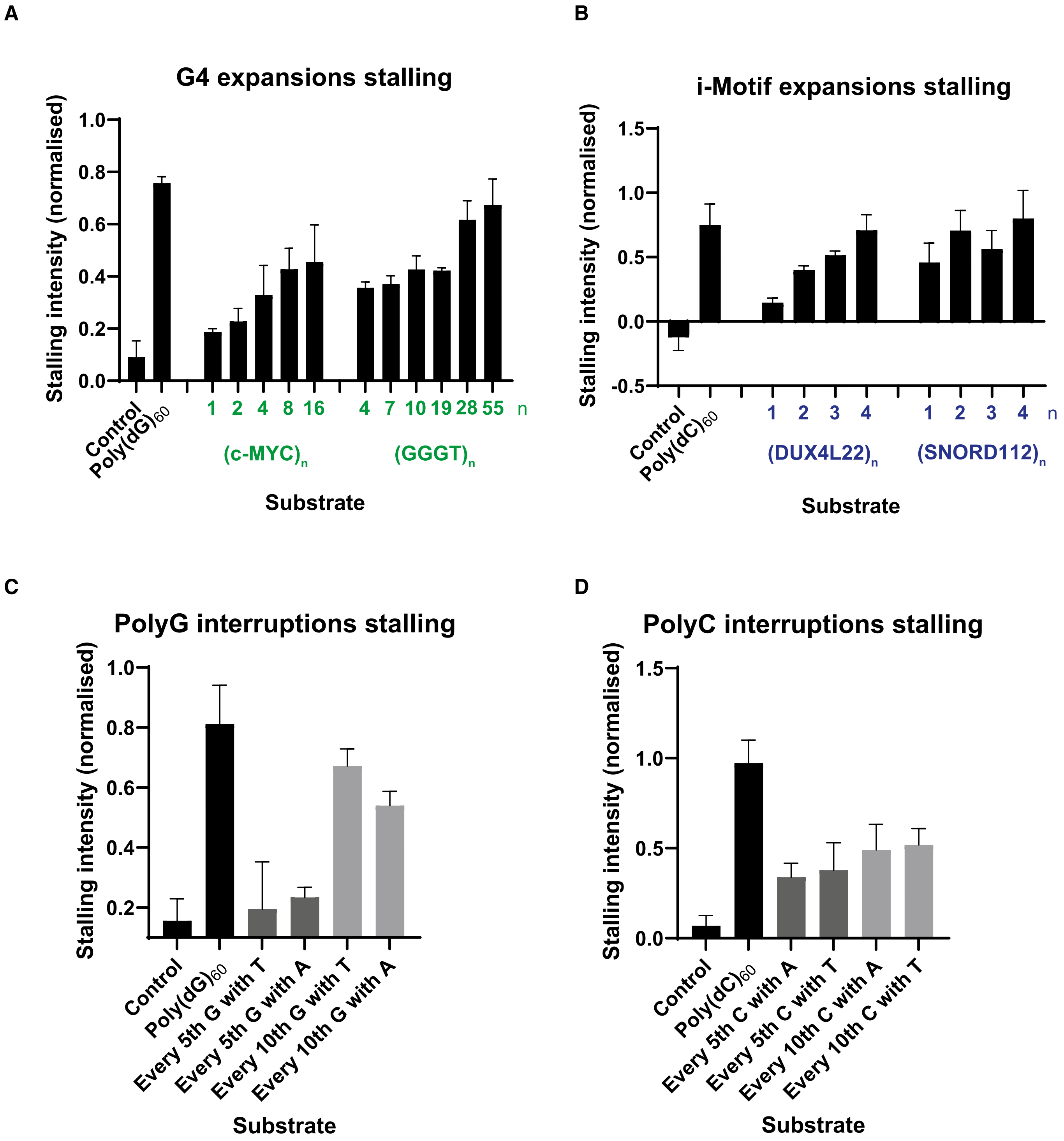

Figure EV3. The effect of increased consecutive quadruplex sequences and interrupting tracts of poly(dG)$_{60}$ or poly(dC)$_{60}$ on replisome stalling.

A–D Quantification of stall intensities from three independent experiments of relevant substrates, as indicated in Fig 2A–D. The 3 kb stalling intensity band was normalised to the intensity of 'leading strand 2' in each lane to account for variation in the efficiencies of reactions for each substrate. The mean stalling intensity is plotted with error bars representing the standard error.

© 2023 The Authors

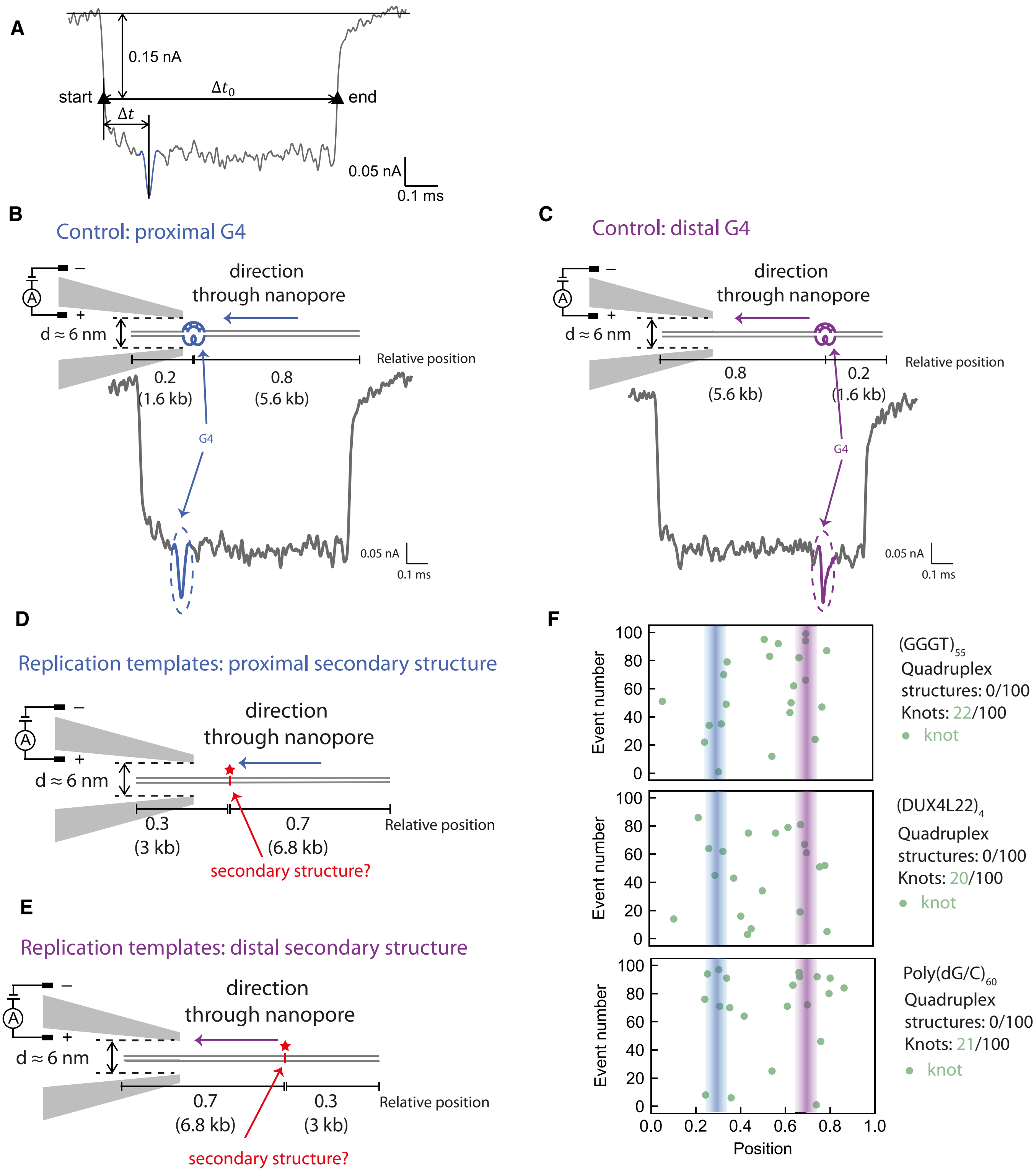

Figure EV4.

© 2023 The Authors

Figure EV4. Single molecule nanopore experiments to detect secondary structures.

A A representative event of our nanopore measurements. The two black triangles, namely the two intersections of the reference line 0.15 nA below the current baseline and the event current trace, mark the start and end of one event. Δt_0 refers to the timescale between the start and end, and Δt refers to the interval between second-level peak and the start.

B, C Schematic of a positive control DNA passing through the nanopore in the direction that positions the G4 proximally (B) or distally (C), and a representative nanopore measurement event. Numbers indicate the proportion through the template the G4 is positioned. The G-quadruplex structure and its corresponding current drop are marked in blue (B) or purple (C).

D, E Schematic of DNA replication templates passing through the nanopore in the direction that positions the potential secondary structures proximally (D) or distally (E). Numbers indicate the proportion through the template the secondary structure-forming sequence is positioned.

F Nanopore measurement results of $(GGGT)_{55}$, $(DUX4L22)_4$ and Poly$(dG/C)_{60}$. Lines indcate expected positions of secondary structures.

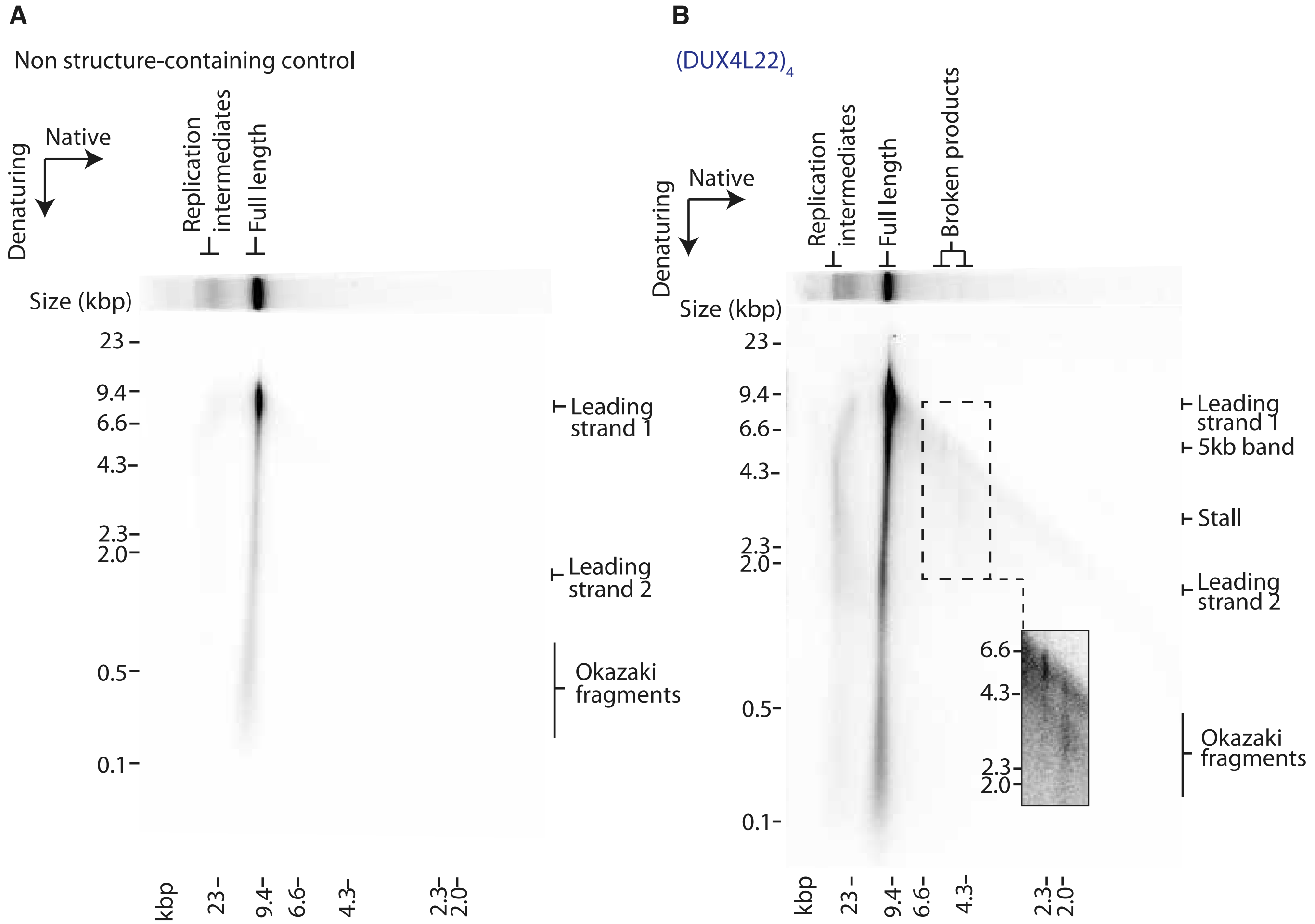

Figure EV5. Analysis of replication products by two-dimensional (2D) gel electrophoresis.

A, B Analysis of replication products of control (A) or iM (B) substrates by 2D gels. Replicated products were run firstly in the native dimension and subsequently in the denaturing dimension.

 © 2023 The Authors
