# Supplementary material for: Replication‐induced DNA secondary structures drive fork uncoupling and breakage
Source: EMBO J. 2023 Oct 2;42(22):e114334. doi: 10.15252/embj.2023114334 (PMC10646557; doi:10.15252/embj.2023114334)
Supplement: Supplementary file 12 [file EMBJ-42-e114334-s008.pdf]

THE EMBO JOURNAL

Replication-induced DNA secondary structures drive fork uncoupling and breakage

Gideon Coster, Sophie Williams, Corella Casas-Delucchi, Federica Raguseo, Dilek Guneri, Yunxuan Li, Masashi Minamino, Emma Fletcher, Joseph Yeeles, Ulrich Keyser, Zoë Waller, and Marco Di Antonio
DOI: 10.15252/embj.2023114334

Corresponding author(s): Gideon Coster (gideon.coster@icr.ac.uk)

Review Timeline:

Submission Date:	20th Apr 23
Editorial Decision:	12th Jun 23
Revision Received:	23rd Aug 23
Editorial Decision:	20th Sep 23
Revision Received:	20th Sep 23
Accepted:	21st Sep 23

Editor: Hartmut Vodermaier

Transaction Report:

(Note: With the exception of the correction of typographical or spelling errors that could be a source of ambiguity, letters and reports are not edited. Depending on transfer agreements, referee reports obtained elsewhere may or may not be included in this compilation. Referee reports are anonymous unless the Referee chooses to sign their reports.)

Dr. Gideon Coster
Institute of Cancer Research
Cancer Biology
237 Fulham Road
London SW3 6JB
United Kingdom

12th Jun 2023

Re: EMBOJ-2023-114334
Replication-induced DNA secondary structures drive fork uncoupling and breakage

Dear Gideon,

Thank you for your patience during the external review of your recent submission, and once more apologies for the slow process. One delayed referee had promised to still send their report before the start of my vacation, but eventually delivered it only a few days after I had left; so that I am only now finally in the position to send you the full set of referee reports, included below.

As you will see, all three referees consider the subject of your work, as well as its findings, important and potentially interesting. We would therefore be happy to pursue a revised manuscript further for EMBO Journal publication, in case you should be able to adequately address a number of specific concerns noted in the three reports.

Since it is our policy to consider only a single round of major revision and therefore important to fully answer to all comments at the time of resubmission, I would invite you to consider the referees' comments together with your coworkers, and to prepare a tentative response letter detailing how each of the raised criticisms/queries might be answered/clarified. On the basis of this response, we could then discuss the requirements for a successful revision already during the early stages of the revision, e.g. via email or a follow-up video call. I should add that we could also offer extension of the default three-months revision period if needed, with our 'scooping protection' (meaning that competing work appearing elsewhere in the meantime will not affect our considerations of your study) remaining of course valid also throughout this extension.

Detailed information on preparing, formatting and uploading a revised manuscript can be found below and in our Guide to Authors. Thank you again for the opportunity to consider this work for The EMBO Journal, and I look forward to hearing from you in due time.

With kind regards,

Hartmut

Hartmut Vodermaier, PhD
Senior Editor, The EMBO Journal
h.vodermaier@embojournal.org

*** PLEASE NOTE: All revised manuscript are subject to initial checks for completeness and adherence to our formatting guidelines. Revisions may be returned to the authors and delayed in their editorial re-evaluation if they fail to comply to the following requirements (see also our Guide to Authors for further information):

1) Every manuscript requires a Data Availability section (even if only stating that no deposited datasets are included). Primary datasets or computer code produced in the current study have to be deposited in appropriate public repositories prior to resubmission, and reviewer access details provided in case that public access is not yet allowed. Further information: embopress.org/page/journal/14602075/authorguide#dataavailability

2) Each figure legend must specify
- size of the scale bars that are mandatory for all micrograph panels
- the statistical test used to generate error bars and P-values
- the type error bars (e.g., S.E.M., S.D.)
- the number (n) and nature (biological or technical replicate) of independent experiments underlying each data point
- Figures may not include error bars for experiments with n<3; scatter plots showing individual data points should be used instead.

3) Revised manuscript text (including main tables, and figure legends for main and EV figures) has to be submitted as editable text file (e.g., .docx format). We encourage highlighting of changes (e.g., via text color) for the referees' reference.

4) Each main and each Expanded View (EV) figure should be uploaded as individual production-quality files (preferably in .eps, .tif, .jpg formats). For suggestions on figure preparation/layout, please refer to our Figure Preparation Guidelines: http://bit.ly/EMBOPressFigurePreparationGuideline

5) Point-by-point response letters should include the original referee comments in full together with your detailed responses to them (and to specific editor requests if applicable), and also be uploaded as editable (e.g., .docx) text files.

6) Please complete our Author Checklist, and make sure that information entered into the checklist is also reflected in the manuscript; the checklist will be available to readers as part of the Review Process File. A download link is found at the top of our Guide to Authors: embopress.org/page/journal/14602075/authorguide

7) All authors listed as (co-)corresponding need to deposit, in their respective author profiles in our submission system, a unique ORCiD identifier linked to their name. Please see our Guide to Authors for detailed instructions.

8) Please note that supplementary information at EMBO Press has been superseded by the 'Expanded View' for inclusion of additional figures, tables, movies or datasets; with up to five EV Figures being typeset and directly accessible in the HTML version of the article. For details and guidance, please refer to: embopress.org/page/journal/14602075/authorguide#expandedview

9) Digital image enhancement is acceptable practice, as long as it accurately represents the original data and conforms to community standards. If a figure has been subjected to significant electronic manipulation, this must be clearly noted in the figure legend and/or the 'Materials and Methods' section. The editors reserve the right to request original versions of figures and the original images that were used to assemble the figure. Finally, we generally encourage uploading of numerical as well as gel/blot image source data; for details see: embopress.org/page/journal/14602075/authorguide#sourcedata

At EMBO Press, we ask authors to provide source data for the main manuscript figures. Our source data coordinator will contact you to discuss which figure panels we would need source data for and will also provide you with helpful tips on how to upload and organize the files.

Further information is available in our Guide For Authors: https://www.embopress.org/page/journal/14602075/authorguide

In the interest of ensuring the conceptual advance provided by the work, we recommend submitting a revision within 3 months (10th Sep 2023). Please discuss the revision progress ahead of this time with the editor if you require more time to complete the revisions. Use the link below to submit your revision:

Link Not Available

Referee #1:

This is an interesting study that shows that individual G4- and i-motif-forming sequences are sufficient to stall reconstituted budding yeast DNA replication machinery in vitro. Using solid-state nanopore sensing approach, the authors found that none of their replication substrates contained pre-formed non-B structures, which led them to conclude that those structures are formed in the course of DNA replication. More specifically, their experimental data show that the replicative helicase readily unwinds both G4 and iM sequences, implying that non-B DNA structures are formed owing to the uncoupling of the CMG helicase and Polε, ultimately blocking the leading strand synthesis. They further found that iM-structures (but not G4-DNA) induce breakage of nascent DNA strands. Finally, forks stalled at G4- and iM-structures are efficiently rescued by DNA-helicase Pif1.

These are substantial and thought-provoking results, which have broad implication for understanding of genome instability caused by replication stalling at natural impediments. I am generally positive about the data and their interpretations, and enjoyed some the authors clever experimental approaches, such as solid-state nanopore sensing or the use of catalytically dead Polε mutant causing Polδ to synthesize both leading and lagging strands . That being said, I have several concerns that the authors may want to address before moving on further.

(1) Many of the stalls caused by G4-DNAs are quite weak, which makes me somewhat skeptical about the validity of their

quantitative analysis. Furthermore, there is a significant variability in stall strengths between different experiments, which is not explained. For example, there is a prominent difference in the intensity of fork stalling at the G60 run between Fig. 1C (very strong) and 1D (quite weak). Also, the differences in fork stalling at G4-motifs and their mutant derivatives (Fig. 1E) do not always strike me as statistically significant. Are they?
The same is even more true for the iM-mediated fork stalling. It is visible for the C60 run and SNORD112, but barely detectable for other motifs, and they also vary strongly between different experiments.
Given these concerns, the authors may want to concentrate on the strongest cases and statistically significant differences in the main text, while moving weaker cases to the supplemental information.
(2) It is not entirely clear to me why the authors conclude that stalling efficiency is determined by the probability of a structure formation rather than its stability. If this is the case and ssDNA window between the CMG and Polε is 16 nt, one would expect the (GGGT)n repeat to cause stronger stalling than the (GGGGT)n repeat. Is this the case based on the data obtained? Also, (GGGGTT)n run should not cause much stalling, even though it forms an exceptionally stable G4-structure.
(3) While the authors claim that no stalling is seen when a G4-motif is on the lagging strand template, I can see stalling in this case in Fig. 2E. Once again, statistical analysis to justify this conclusion is lacking.
(4) I am still puzzled why would iM-structures be formed at pH 7.5 in the author's experiments, given that every other cytosine has to be protonated in order for them to form. Would these weak iM-mediated stalls get stronger upon a decrease in pH to 7.0 or 6.5 (which is, in fact, closer to the pH in yeast nuclei)? Also, could iM-mediated stalls in the leading strand template simply reflect G4-mediated stalls in the lagging strand template?
(5) I feel that intrinsic repriming is far less exotic possibility than replication-induced breakage of iM-structures.
(6) Given that Pif1 is a 5'-to-3' DNA helicase, do the authors think that it moves along the leading strand template in the opposite to CMG direction, or that it moves along the lagging strand template in the same direction as the fork?
(7) Table 1 is nowhere to be found.

Minor Comment:
(1) Hairpin-forming repeat cause replication stalling in vivo not just in yeast, but also in bacteria and human cells.

Referee #2:

The manuscript uses a multidisciplinary approach to address how DNA secondary structures formed from G4 motifs or i-motifs affect DNA replication. The use of the purified leading-strand replication system is a major advance that allows new insights to be drawn. The results are clear that replication leads to structure formation, and that some structures stall DNA replication at the structure to cause helicase-polymerase uncoupling. Evidence of replication-induced double-strand breaks is presented for some motifs. Rescue of the stalled fork is provided by Pif1 helicase but not by two related enzymes. Overall, the study adds considerably to our understanding of how alternative DNA structures at some G4 and i-motifs impacts replication.

Major concerns.
1. The experiments in Fig2EF seem to be of limited benefit. 2E shows no effect, and 2F gave results that require speculative interpretation. Suggest dropping this experiment until lagging strand synthesis can be evaluated directly.

2. I was not convinced by the data in Fig 5C. It is very hard to see the 1.5 and 3-4 kb bands referred to in the text, which weakens the argument that they map to the leading nascent strands at the break site.

Minor points.
1. Page 3, middle paragraph. I suggest spelling out the iM consensus so that the format matches that for the G4 consensus on the previous page. (C5N1-19C5N1-19C5N1-19C5). This format is also clearer to the reader. The subsequent sentence ("The thermal stability of iMs...") does not make sense, since it states the stability can be measured via calculation; "measured" implies experimental readout, not calculation.

2. Page 13, middle paragraph. The text describing Fig 5C states "Intriguingly, 2D gel analysis maps the 5 kb band to the products on the native dimension as broken". I don't see why this is intriguing. Explain.

3. Table 1 needs modification to indicate that the Tm values were measured by the technique referred to in the main text page 6.

4. All four tables in the merged PDF were problematic. Tables 1 and 4 do not appear at all, and the errant formatting for Tables 2 and 3 made them impossible to read. It was necessary to view the individual tables in Excel format.

Referee #3:

The manuscript from Williams et al is very clearly structures and very well written. In this manuscript they use detailed biochemical analysis to monitor replication stalling of the leading strand by G4s and iMotifs. Using purified proteins from yeast they monitored length and strength of pausing and which subunit is connected to this stall. The Experiments are done with great care and the topic is interesting although not novel.

Major points
1. They characterized and cloned multiple different G4s into their plasmid constructs. In a very elegant assay they have monitored replication stalling at G4s. However they are all very stable and not from yeast. Did they test also yeast specific G4s, to exclude that the stalling to non yeast once, is not due to the fact that in yeast very little very stable G4s form? Also CMG unwinding should be done on yeast endogenous G4s, to really detect if CMG is not able to unwind G4 that are seen "normally" by the CMG.
2. I am not sure if iM have been detected in yeast. Do they exist in bakers yeast? Please include this information in the well written introduction.
3. How do the authors exclude that the nanopore sequencing is not altering the G4 or iM status? As the point that there are no prefolded G4s in the construct is very important for the subsequent paper, an alternative assay would be good. THave they also performed an alternative strategy (e.g. BG4 ChIP, or a light up probe like NMM)
4. They observed break products within the iM (5kb product), it would be very interesting to test where and why the iM leads to this break product. Maybe sequencing of this product would help to determine how the break forms in this region. Also it is essential to discuss how iM would lead to breaks and how this would be reflect replication and genome stability
5. The experiment they used helicase addition to the stalling are very good. As yeast have more helicases than Sgs1, Chl1 and Pif1 the selection of these three is not clear to me. Hrq1, Rrm3 are potential candidates that also are implicated in replication progression and G4s are other candidates to test (Rrm3 full length is not purifiable yet, but the truncated version has been purified)

Replication-induced secondary structures drive fork uncoupling and breakage: reviewer comments

We thank all three referees for taking the time to review our manuscript and we appreciate all the useful feedback we have received. We have taken onboard the valuable comments outlined and taken the time to update our work and manuscript accordingly, which we feel has significantly enhanced our manuscript. We outline below how we have addressed those points raised, both experimentally and in written form.

Referee #1 (Remarks to author):

This is an interesting study that shows that individual G4- and i-motif-forming sequences are sufficient to stall reconstituted budding yeast DNA replication machinery in vitro. Using solid-state nanopore sensing approach, the authors found that none of their replication substrates contained pre-formed non-B structures, which led them to conclude that those structures are formed in the course of DNA replication. More specifically, their experimental data show that the replicative helicase readily unwinds both G4 and iM sequences, implying that non-B DNA structures are formed owing to the uncoupling of the CMG helicase and Polε, ultimately blocking the leading strand synthesis. They further found that iM-structures (but not G4-DNA) induce breakage of nascent DNA strands. Finally, forks stalled at G4- and iM-structures are efficiently rescued by DNA-helicase Pif1.

These are substantial and thought-provoking results, which have broad implication for understanding of genome instability caused by replication stalling at natural impediments. I am generally positive about the data and their interpretations, and enjoyed some the authors clever experimental approaches, such as solid-state nanopore sensing or the use of catalytically dead Polε mutant causing Polδ to synthesize both leading and lagging strands. That being said, I have several concerns that the authors may want to address before moving on further.

Major points

(1) Many of the stalls caused by G4-DNAs are quite weak, which makes me somewhat skeptical about the validity of their quantitative analysis. Furthermore, there is a significant variability in stall strengths between different experiments, which is not explained. For example, there is a prominent difference in the intensity of fork stalling at the G60 run between Fig. 1C (very strong) and 1D (quite weak). Also, the differences in fork stalling at G4-motifs and their mutant derivatives (Fig. 1E) do not always strike me as statistically significant. Are they?

The same is even more true for the iM-mediated fork stalling. It is visible for the C60 run and SNORD112, but barely detectable for other motifs, and they also vary strongly between different experiments.

Given these concerns, the authors may want to concentrate on the strongest cases and statistically significant differences in the main text, while moving weaker cases to the supplemental information.

We thank the reviewer for acknowledging the relevance of our work and the potential implications for the genome stability field. The reviewer has noted the variability between stalling intensities at G4s and iMs in different experiments. We thank the reviewer for highlighting this and acknowledge that in some experiments the stalls appear stronger than in others. This is because the *in vitro* replication assay is a highly complex and sensitive assay which produces results that are inherently variable. Whilst we cannot change this, we acknowledge that it may make the levels

of stalling difficult to interpret. However, sequences that stall replication do so consistently, and quantifications of stalling are always compared to the control template and the 1.5 kb left leading strand to account for variability between assays and templates. To acknowledge variability we have changed the text in the manuscript as follows (Page 7 paragraph 2):

> "The efficiency of replication reactions is inherently variable between different experiments and different templates. This can result in variability in the stalling intensities observed for any given sequence. To account for this, the intensity of the 3kb stall band is quantified from three independent experiments and normalised to the intensity of the 1.5 kb band (**Fig. S2A**)"

We have also repeated the experiment in Figure 1C to test the effects of a single iM on replication progression. We have removed the G4-forming sequences from this figure to allow the iM stalls to be visualised more easily and updated the figure accordingly.

The reviewer also suggests moving substrates that cause weaker stalls to the supplementary information. Whilst we appreciate the fact that some sequences have no effect or induce very weak stalls, we feel that keeping them is informative. This is because it adds to the evidence that a more stable structure induces stronger replisome stalling. It also argues that structure formation is indeed the cause of replication stalling as weaker structures (such as PIM1 or 4xTTAGGG) do not cause replication stalling.

The reviewer also points out that the differences in stalling between structure-forming motifs and their mutant derivatives is not always clear. We believe a major reason for this difficulty is the inherently weak stalling induced by a single iM-forming sequence. Stalling by two consecutive iM-forming sequences is stronger. We therefore also expanded mutant sequences to 2x. This produced a clearer difference in stalling between the WT and mutated sequences. Accordingly, we have updated Figure 1F and 1G with the expanded iM sequences. We have updated the manuscript as follows (page 8 paragraph 1):

> "Testing the effect of mutations on individual iM-forming sequences was challenging, as stalling at a single iM was generally weaker than that observed at a single G4. We therefore tested the effect of two consecutive iMs and observed stronger replisome stalling (Fig. 1F), which was sufficiently strong for analysis of mutants. When the iM-forming sequences DUX4L22 and SNORD112 were disrupted for their structure-forming ability (**Fig. S3B**), stalling was reduced (**Fig. 1F, 1G**). This was less evident for DUX4L22, which is reflected in the fact that the mutations have a greater effect on the transitional pH and thermal stability of SNORD112, with a small effect on DUX4L22 (**Fig. S3D, S3F, S3G** and Table 2)."

(2) It is not entirely clear to me why the authors conclude that stalling efficiency is determined by the probability of a structure formation rather than its stability. If this is the case and ssDNA window between the CMG and Polε is 16 nt, one would expect the (GGGT)n repeat to cause stronger stalling than the (GGGGT)n repeat. Is this the case based on the data obtained? Also, (GGGGTT)n run should not cause much stalling, even though it forms an exceptionally stable G4-structure.

We thank the reviewer for bringing this issue to our attention. Whilst we believe probability of structure formation plays a role in the likelihood of replisome stalling,

we believe this is also affected by the stability of the structure. In other words, a more thermally stable structure is more likely to induce replisome arrest (as demonstrated in Figure 1). In addition to this, if a structure is more likely to fold during replication (either due to increased windows of opportunity for structure formation as in polyd(G)60 or due to faster folding kinetics) then it is more likely to stall the replisome. To clarify that both stability and probability play a role in inducing replication staling, we have updated the text as follows:

> Page 8 paragraph 3: "These observations suggest that in addition to structure stability (**Fig. 1**), stalling efficiency is also dictated by the probability of secondary structure formation. To test this possibility, we interrupted the poly(dG)$_{60}$ sequence such that it could still support G4-formation but constrained to specific G-tracts. …We conclude that stalling efficiency is determined not only by structure stability, but also by the probability of structure formation."

> Page 19 paragraph 1: "Folding kinetics usually correlate with structure stability, which may also contribute to the fact that both structure stability and probability of formation play a role in inducing replication stalling."

The reviewer also raised the question as to whether (GGGT)n would cause stronger stalling than (GGGGT)n and (GGGGTT)n. Based on the amount of ssDNA required to be exposed between CMG and pol ε for a structure to be able to form, together with the predicted thermal stability of the structures, one would predict that the intensity of stalling would be as follows: **GGGT > GGGGT > GGGGTT > GGGTTT.** To test this, we cloned these substrates and tested their effects on replication. We find strong stalling induced by GGGT, and weaker stalling induced by longer repeats. This confirmed our hypothesis and strengthens the argument that stalling is determined by both the likelihood of structure formation and structure stability.

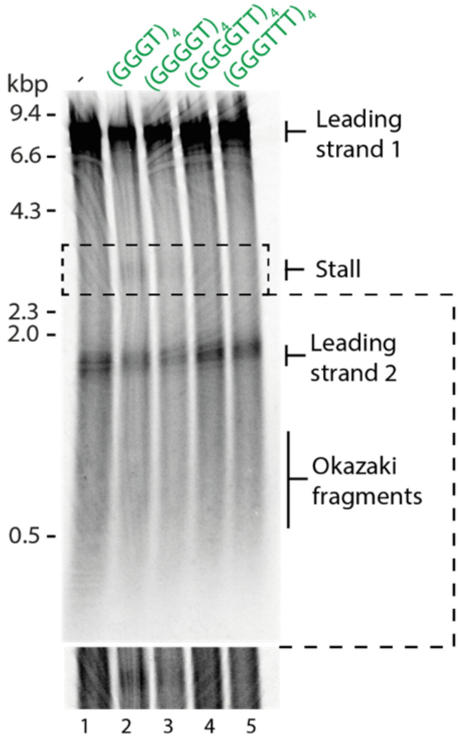

(3) While the authors claim that no stalling is seen when a G4-motif is on the lagging strand template, I can see stalling in this case in Fig. 2E. Once again, statistical analysis to justify this conclusion is lacking.

To clarify our conclusions regarding the effects of orientation of the G4 or iM relative to the replication origin, we have repeated the experiment 3 times and quantified the intensity of stalling induced:

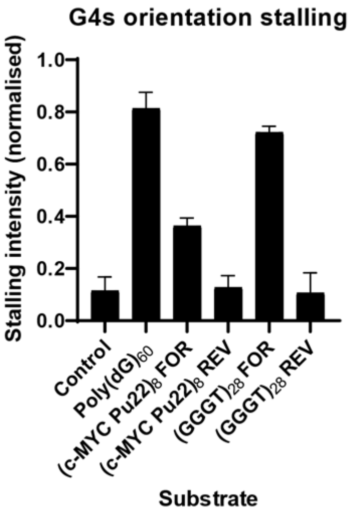

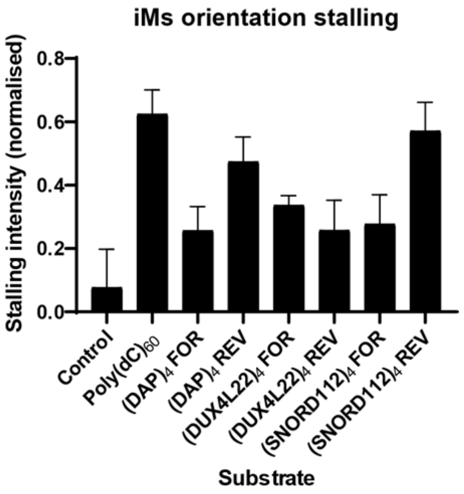

Given the quantifications shown above, we believe that it is clear that G4s in the forward orientation (on the leading strand template) induce leading strand replication stalling, but do not affect replisome progression when found in the reverse orientation (on the lagging strand template). In contrast, iMs found in both the forward and reverse orientation impede replisome progression. We believe that this is because the complementary sequence to iMs would form a strong G4 on the leading strand template which in turn inhibits replication progression.
We acknowledge that this figure may have caused some confusion to the reader in terms of the set up and interpretation, as although we are assessing the effects of the G4 or the iM on the leading versus lagging strand template, we are only studying leading strand progression. To clarify this figure and make it easier to interpret, we have updated the figures (Fig. 2E and 2F) with new labels (forward and reverse) and accompanying schematics.

(4) I am still puzzled why would iM-structures be formed at pH 7.5 in the author's experiments, given that every other cytosine has to be protonated in order for them to form. Would these weak iM-mediated stalls get stronger upon a decrease in pH to 7.0 or 6.5 (which is, in fact, closer to the pH in yeast nuclei)? Also, could iM-mediated stalls in the leading strand template simply reflect G4-mediated stalls in the lagging strand template?

We thank the reviewer for raising the question of pH, as this is known to have a strong impact on the formation and stability of iM structures. As such, we also questioned whether a lower pH would result in more stable iM structures and increased replication stalling. However, when we decreased the pH of the assay to 6 (versus 7.6), we saw no significant effect on replisome stalling. We tried this experiment several times and a representative example can be seen below:

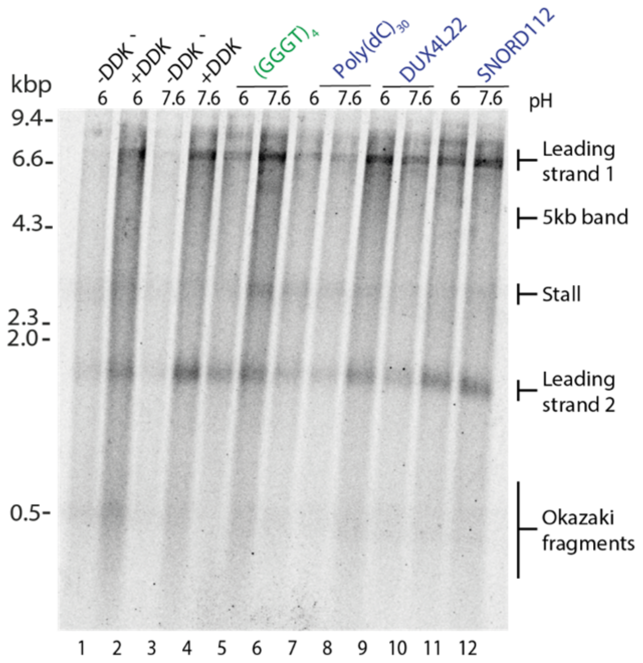

Although we were somewhat surprised that lowering the pH had no effect on iM-induced replication stalling, we are confident that these iM structures can form at pH 7.6 based on our biophysical data and the fact that the iMs chosen were all selected as they have been shown to form at neutral pH [1].

The reviewer also raised the question as to whether iM-mediated stalls in the leading strand template could reflect G4-mediated stalls on the lagging strand template. If this were the case, then positioning a strong G4-forming sequence on the lagging strand would induce leading strand stalls. However, Fig 2E shows that a strong G4 on the lagging strand does not induce leading strand stalls. We therefore favour an interpretation whereby the stalling induced by iM-forming sequences on the leading strand is caused by a leading strand iM structure.

(5) I feel that intrinsic repriming is far less exotic possibility than replication-induced breakage of iM-structures.

We agree with the reviewer that iM-induced breakage is an exciting finding. We have considered both intrinsic repriming and nascent DNA breakage at iMs. Currently, our native and 2D gels (Figure 5) support the latter. However, we have not excluded the possibility of repriming. We do, however, note that intrinsic repriming downstream of a leading strand lesion is very inefficient [2].

(6) Given that Pif1 is a 5'-to-3' DNA helicase, do the authors think that it moves along the leading strand template in the opposite to CMG direction, or that it moves along the lagging strand template in the same direction as the fork?

Our working model is that structures form on the leading strand template behind CMG and that Pif1 moves along the leading strand template in the opposite direction to CMG to unwind structures and allow DNA synthesis to resume (see model in Fig. 6C).

(7) Table 1 is nowhere to be found.

We apologise for the fact that there was a formatting issue upon submission. We have now converted tables 1 and 2 into a Word table (from Excel) which should resolve formatting issues. However, tables 3 and 4 contain too much information to be converted to Word tables, and are therefore accessible as Excel spreadsheets.

Minor Comment:
(1) Hairpin-forming repeat cause replication stalling in vivo not just in yeast, but also in bacteria and human cells.

We thank the reviewer for raising this to our attention and have updated the introduction as follows (page 2 paragraph 2):

"Examples of well-characterised secondary structures include hairpin[3, 4], G-quadruplex (G4)[5] and intercalated-Motif (iM)[6] structures, which are thought to act as barriers to replication. For example, hairpin-forming repeats cause replication stalling in bacteria, yeast, and human cells[7]."

Referee #2 (Remarks to Author):

The manuscript uses a multidisciplinary approach to address how DNA secondary structures formed from G4 motifs or i-motifs affect DNA replication. The use of the purified leading-strand replication system is a major advance that allows new insights to be drawn. The results are clear that replication leads to structure formation, and that some structures stall DNA replication at the structure to cause helicase-polymerase uncoupling. Evidence of replication-induced double-strand breaks is presented for some motifs. Rescue of the stalled fork is provided by Pif1 helicase but not by two related enzymes. Overall, the study adds considerably to our understanding of how alternative DNA structures at some G4 and i-motifs impacts replication.

Major concerns.
1. The experiments in Fig2EF seem to be of limited benefit. 2E shows no effect, and 2F gave results that require speculative interpretation. Suggest dropping this experiment until lagging strand synthesis can be evaluated directly.

We thank the reviewer for acknowledging the relevance and importance of our work and hope it will provide new insights into how DNA secondary structures affect genome integrity. This reviewer, like reviewer #1, has raised Figures 2E and 2F and questioned their relevance. As noted above, we appreciate that this experiment may have caused some confusion as although we are positioning G4s or iMs on the lagging strand template, we are only looking at the effects on leading strand replication. To address this, we have updated the figures (Fig. 2E and 2F) with new labels (forward and reverse) and accompanying schematics.

In addition to this, we have updated the text in the manuscript as follows (Page 10, paragraphs 2 and 3):

"Having established that replication is stalled due to a quadruplex-forming sequence on the leading strand template, we next sought to determine how a quadruplex-forming sequence on the lagging strand template affects leading strand replication. To this end, we cloned G4-forming sequences in the

forward orientation (leading strand template) or reverse orientation (lagging strand template) and observed leading strand replication products (Fig. 2E). It is important to note that although we have cloned structure-forming sequences on the lagging strand template (reverse), we did not include factors required for Okazaki fragment maturation, and as such we only observed the effects on leading strand replication. While leading strand stalling was observed when the G4-forming sequence was in the forward orientation, no stalling was seen when in the reverse orientation (Fig. 2E, compare lanes 3 and 5 to lanes 4 and 6). Although we did not analyse lagging strand synthesis, previous work has demonstrated that the replication machinery skips over a lagging strand G4, resulting in a small gap, about the size of an Okazaki fragment, on the nascent lagging strand (49).

We performed similar experiments to determine how an iM-forming sequence on the lagging strand template affects leading strand synthesis. Interestingly, leading strand stalling occurred in both orientations (forward and reverse) (Fig. 2F). Given the shorter consensus sequence of stable G4s relative to iMs, we speculate that the stalling observed in the reversed orientation in this scenario is due to G4 formation by the complementary G-rich sequence on the leading strand template. In contrast, the C-rich sequences which are complementary to the G4-forming sequences in Figure 2E are not able to form very stable iM structures (Table 2, both have a low pHT ~5.8). This may explain why we do not observe leading strand replication stalling when these sequences are positioned on the leading strand template (Fig. 2E, lanes 4 and 6)."

We hope this will provide clarity to the results and the interpretations we have made.

2. I was not convinced by the data in Fig 5C. It is very hard to see the 1.5 and 3-4 kb bands referred to in the text, which weakens the argument that they map to the leading nascent strands at the break site.

We thank the reviewer for highlighting the fact that the 1.5 and 3-4 kb bands are difficult to see in Figure 5C. We note that these bands represent a small proportion of replication products and as such are sometimes difficult to visualise, especially upon processing by 2D gels where some material can be lost in each dimension. To ensure these bands are visible to the reader, we have enhanced the contrast of the section containing the 1.5 and 3-4 kb bands and included this as an insert in Figure 5C. On top of this, we have updated the text in the manuscript to focus more on the 5 kb band, which is more important for our conclusions, instead of these smaller bands (page 13, paragraph 3):

"2D gel analysis maps the 5 kb band to the products identified on the native dimension as broken (Fig. 5C). We also observed a weaker population of products in the denaturing dimension corresponding to the second faster migrating broken product on the native gel (Fig. 5C). Importantly, these bands mapped to the same positions observed with the simulated broken products (Fig. 5D), were absent from the empty vector (Fig. S7A) and were also observed with a different iM-forming sequence (Fig. S7B)."

Minor points.

1. Page 3, middle paragraph. I suggest spelling out the iM consensus so that the format matches that for the G4 consensus on the previous page. (C5N1-19C5N1-19C5N1-19C5).

This format is also clearer to the reader. The subsequent sentence ("The thermal stability of iMs...") does not make sense, since it states the stability can be measured via calculation; "measured" implies experimental readout, not calculation.

We thank the reviewer for this suggestion and have updated the manuscript accordingly (page 3 paragraph 2):

"However, recent studies have shown that certain iMs can form at neutral pH[1], requiring longer tracts of cytosines with a proposed consensus of four consecutive tracts of at least five cytosines, separated by one to nineteen non-C nucleotides ($C_5N_{1-19}C_5N_{1-19}C_5N_{1-19}C_5$)[1]. The thermal stability of iMs can be determined similarly to G4s by measuring the melting temperature of the structure."

2. Page 13, middle paragraph. The text describing Fig 5C states "Intriguingly, 2D gel analysis maps the 5 kb band to the products on the native dimension as broken". I don't see why this is intriguing. Explain.

We have updated the text to remove the word 'intriguingly' as follows (page 13, paragraph 3):

"Full-length products consisted mostly of full-length leading strands 1 and 2 and Okazaki fragments. 2D gel analysis maps the 5 kb band to the products identified on the native dimension as broken (Fig. 5C)."

3. Table 1 needs modification to indicate that the Tm values were measured by the technique referred to in the main text page 6.

We thank the reviewer for raising this issue and have updated the table accordingly.

4. All four tables in the merged PDF were problematic. Tables 1 and 4 do not appear at all, and the errant formatting for Tables 2 and 3 made them impossible to read. It was necessary to view the individual tables in Excel format.

Similar to reviewer #1, we thank the reviewer for raising this to our attention and apologise for the fact that there was a formatting issue upon submission. We have now converted Tables 1 and 2 into Word tables (from Excel) which should resolve formatting issues. However, tables 3 and 4 contain too much information to be converted to Word tables, and are therefore accessible as Excel spreadsheets.

Referee #3 (Remarks to Author):

The manuscript from Williams et al is very clearly structures and very well written. In this manuscript they use detailed biochemical analysis to monitor replication stalling of the leading strand by G4s and iMotifs. Using purified proteins from yeast they monitored length and strength of pausing and which subunit is connected to this stall. The Experiments are done with great care and the topic is interesting although not novel.

Major points

1. They characterized and cloned multiple different G4s into their plasmid constructs. In a very elegant assay they have monitored replication stalling at G4s. However they are all very stable and not from yeast. Did they test also yeast specific G4s, to exclude that the stalling

to non yeast once, is not due to the fact that in yeast very little very stable G4s form? Also CMG unwinding should be done on yeast endogenous G4s, to really detect if CMG is not able to unwind G4 that are seen "normally" by the CMG.

We thank the reviewer for acknowledging the clarity and relevance of our work. The reviewer has raised the question as to whether endogenous yeast G4s also stall the eukaryotic replisome. To address this, we cloned and tested more substrates containing endogenous yeast G4 sequences. The G4 sequences we selected were all based on Pif1 ChIP experiments which identified G4 sequences in the yeast genome that bind Pif1 [8]. A representative test of these substrates is shown below:

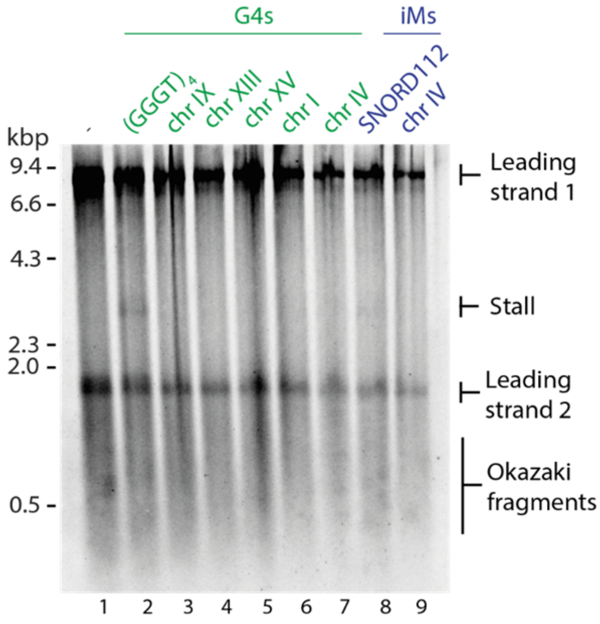

Here, we observe little/no replication stalling at the yeast G4s tested. Although these sequences are all predicted to form G4s, we predict that they would form weaker structures than (GGGT)4 due to the fact they have longer loops composed of more adenine residues:

G4	Sequence (5’-3’)
Chr IX	GGGTACGGTGGGTAATAAGGGAAGGTATCGGG
Chr XIII	GGGGTGGTGCTTCAGCCTGGGGTAACAAATCAAGTTGGGGCGGTGCATCCACTTGGGCGTCGGG
Chr XV	GGGTTTGTTGCAGACTGTGTCGAGGGAATTTCAGGAATTGGGAATGGAAATGGATTGCTTTGGGG
Chr I	GGGGTGGGGTGTTACTGATGAGAATTGGGGGAATTTTGAGATAATTGTTGGG
Chr IV	GGGGAGGGGAAGGGGAGGGG

It is perhaps unsurprising that these sequences induce weaker/no replisome stalling than the G4 substrates we have tested here. Despite this, we do believe our results are representative of the effects of G4s on eukaryotic replisomes. Although we are using purified budding yeast proteins, replication is highly conserved across eukaryotes from yeast to human. Indeed, the reconstituted budding yeast replication system has been used by many different labs to study important aspects of eukaryotic DNA replication.

2. I am not sure if iM have been detected in yeast. Do they exist in bakers yeast? Please include this information in the well written introduction.

We thank the author for raising the question of iMs in budding yeast. Previous work has described a sequence on chromosome IV (coordinates 1242544–1242567) of S. cerevisiae that can fold into an iM [9]:

**iM**	**Sequence (5'-3')**
Chr IV	TT**CCCC**T**CCCC**TT**CCCC**T**CCCC**TC

We have now included this sequence in our introduction as follows:

"Similar to G4s, a longer tract of Cs and a shorter loop length creates a more stable iM. In contrast to G4s, iMs can only adopt an anti-parallel conformation. Although not highly prevalent in the yeast genome, a previous study has described a sequence on chromosome IV of *S. cerevisiae* that can fold into an iM structure[9]. In addition to this, iMs can be found at transcription start sites, and in the promoters of oncogenes such as *BCL2*[10], *HRAS*[11], *KRAS*[12], *c-MYC*[13] and *VEGF*[14]."

We have cloned and tested this sequence for its effects on replisome progression. As shown above (in point 1), we observed no effect on replisome progression. We speculate that this may be because it might not fold into as strong an iM as the human ones we have tested (because there are only 4 tracts of Cs rather than 5).

3. How do the authors exclude that the nanopore sequencing is not altering the G4 or iM status? As the point that there are no prefolded G4s in the construct is very important for the subsequent paper, an alternative assay would be good. THave they also performed an alternative strategy (e.g. BG4 ChIP, or a light up probe like NMM).

We thank the reviewer for raising this question. The nanopore experiments performed here to detect DNA secondary structures do not involve unwinding or separating the dsDNA into ssDNA as they make use of pores with a larger diameter than that which is typically used for nanopore sequencing. As such we do not envisage any effects on pre-existing G4 or iM structures (this is also demonstrated by our positive control).

We have attempted to detect G4s or iMs by alternative methods (dot blots using BG4 and 1H6, NMM and Quinaldine Red, biotin labelled Phen-DC3). Although these were all useful to detect G4s in the context of short oligos, they proved to be not specific and sensitive enough to detect G4s in the context of large amounts of plasmid/duplex DNA.

4. They observed break products within the iM (5kb product), it would be very interesting to test where and why the iM leads to this break product. Maybe sequencing of this product would help to determine how the break forms in this region. Also it is essential to discuss how iM would lead to breaks and how this would be reflect replication and genome stability

We thank the reviewer for acknowledging the importance of DNA breakage at sites of iMs. Whilst we are very interested as to how this process occurs, we believe that it is beyond the scope of this project as it would require extensive experiments to uncover the mechanism of iM-induced DNA breakage. This will be a subject of future studies.

5. The experiment they used helicase addition to the stalling are very good. As yeast have more helicases than Sgs1, Chl1 and Pif1 the selection of these three is not clear to me. Hrq1, Rrm3 are potential candidates that also are implicated in replication progression and G4s are other candidates to test (Rrm3 full length is not purifiable yet, but the truncated version has been purified)

We thank the reviewer for bringing to our attention alternative G4-unwinding helicases that may be able to rescue replisome stalling at G4s and iMs. We have obtained purified full length Rrm3 from Dr. Tom Deegan (University of Edinburgh, UK) and tested its ability to rescue replisome stalling. In contrast to Pif1, we observed no rescue of stalling at G4s or iMs in the presence of Rrm3.
We also obtained constructs for purification of wildtype Hrq1 and a helicase-dead mutant (K318A) from Dr. Matthew L. Bochman (Indiana University, USA) which we subsequently expressed, purified and tested its ability to rescue stalling at G4s and iMs. Similarly, we observed no rescue of G4 or iM stalling. We have confirmed both helicases were active in a helicase assay on model substrates and can therefore conclude the lack of rescue observed is not due to a defect in helicase activity.
Finally, we have also tried to test the Srs2 helicase (functional homolog of RTEL1). Despite multiple attempts at expression and purification using different protocols, we were unable to obtain an enzyme preparation that was active on model substrates.

We have included these as new supplementary figures (Fig. S10E, S10F, S10I, S10J) and updated the manuscript accordingly:

Page 14, paragraph 4: “However, there are additional helicases that bind and unwind G4 structures both *in vitro* and *in vivo*, such as Rrm3, Sgs1, Hrq1 and Chl1. Rrm3 is another yeast Pif1-family helicase with a high sequence and functional similarity to Pif1 [15]. Like Pif1, Rrm3 is a 5’-3’ helicase which has overlapping functions with Pif1 and helps the replisome bypass barriers such as at tRNA promoters and telomeric DNA [16, 17]. Sgs1 is a 3’-5’ RecQ family helicase shown to preferentially unwind G4 DNA and is the yeast homolog of the BLM and WRN helicases [18]. Similarly, Hrq1 is the yeast homolog of another RecQ helicase, RecQ4. RecQ4 is one of the five RecQ helicases found in humans, with 3’-5’ polarity. It functions in telomere maintenance [19] and has been shown to bind and unwind G4 structures [20]. Chl1 is the yeast homolog of human ChlR1 (also called DDX11). ChlR1 is a 5’-3’ helicase that directly unwinds G4 structures and is proposed to help process G4s during DNA replication [21, 22]..”

Page 15, paragraph 1: “Neither Sgs1 (**Fig. S10A, S10B**), nor Chl1 (**Fig. S10C, S10D**), nor Rrm3 (**Fig. S10E**), nor Hrq1 (**Fig. S10F**) were able to rescue stalling at either G4 or iM-forming sequences. Importantly, these enzymes were active on a model substrate, although Rrm3 and Hrq1 displayed only partial unwinding activity (**Fig. S10G, S10H, S10I, S10J**).”

Page 20, paragraph 3: “Interestingly, none of the other helicases tested were able to rescue fork stalling at G4s or iMs, despite their demonstrated ability to unwind G4s[18, 20, 21]. This may reflect the fact that these helicases show a preference for certain types of G4s. For example, Chl1 has been shown to have stronger unwinding activity on anti-parallel G4s[21], while the G4 sequences we tested all form parallel G4s which is a more common conformation. Similarly, the fact that Rrm3 was unable to rescue stalling at G4s despite its relation to Pif1 may reflect the fact that these helicases often have different functions despite binding the same substrates [15]. This may

also explain the large number of quadruplex-unwinding helicases that appear to have some level of redundancy, as they may each have a role in resolving structures in different scenarios.”

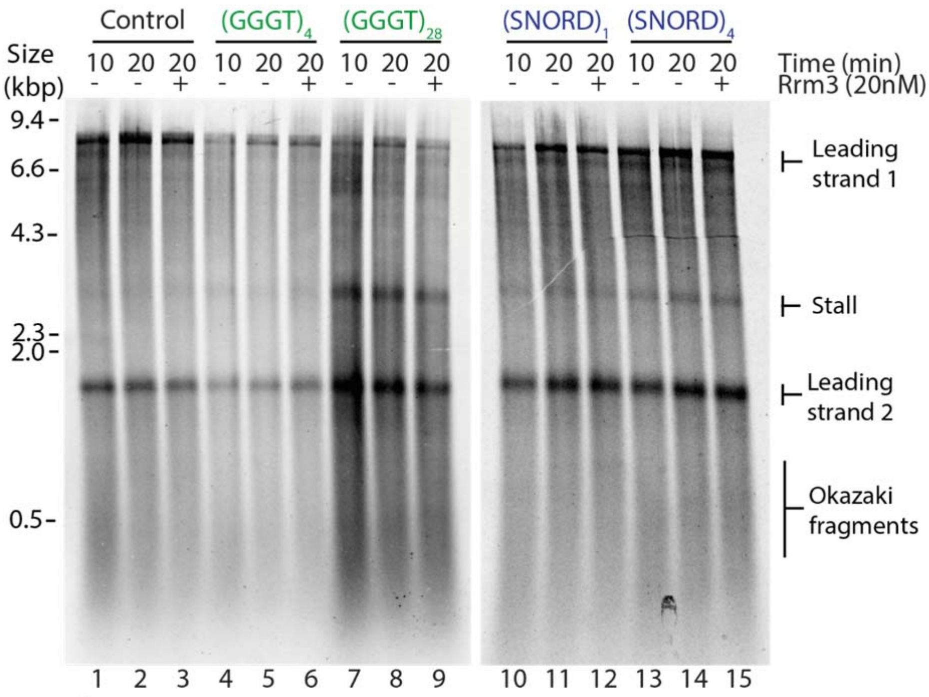

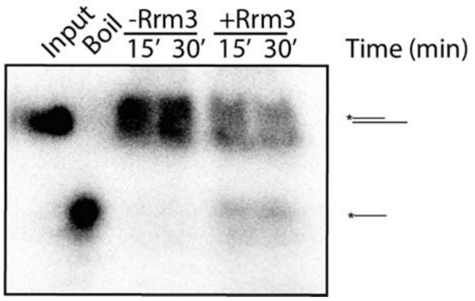

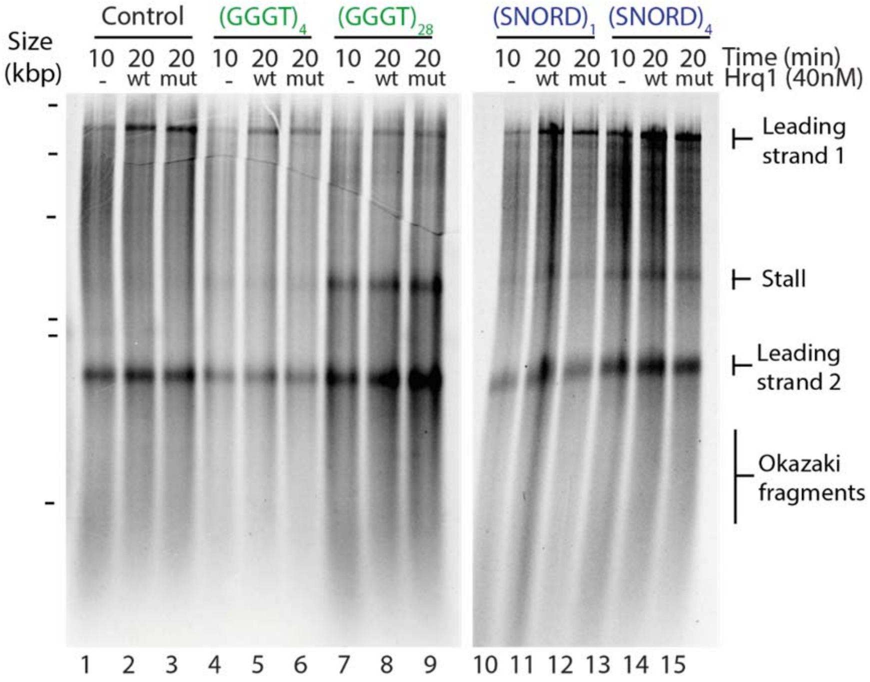

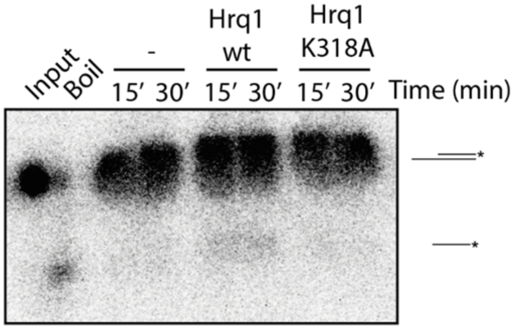

1. Wright, E.P., J.L. Huppert, and Z.A.E. Waller, *Identification of multiple genomic DNA sequences which form i-motif structures at neutral pH.* Nucleic Acids Res, 2017. **45**(6): p. 2951-2959.
2. Taylor, M.R.G. and J.T.P. Yeeles, *The Initial Response of a Eukaryotic Replisome to DNA Damage.* Mol Cell, 2018. **70**(6): p. 1067-1080 e12.
3. Gacy, A.M., et al., *Trinucleotide repeats that expand in human disease form hairpin structures in vitro.* Cell, 1995. **81**(4): p. 533-40.
4. Nadel, Y., P. Weisman-Shomer, and M. Fry, *The fragile X syndrome single strand d(CGG)n nucleotide repeats readily fold back to form unimolecular hairpin structures.* J Biol Chem, 1995. **270**(48): p. 28970-7.
5. Fry, M. and L.A. Loeb, *The fragile X syndrome d(CGG)n nucleotide repeats form a stable tetrahelical structure.* Proc Natl Acad Sci U S A, 1994. **91**(11): p. 4950-4.
6. Gehring, K., J.L. Leroy, and M. Gueron, *A tetrameric DNA structure with protonated cytosine.cytosine base pairs.* Nature, 1993. **363**(6429): p. 561-5.
7. Voineagu, I., et al., *Replication stalling at unstable inverted repeats: interplay between DNA hairpins and fork stabilizing proteins.* Proc Natl Acad Sci U S A, 2008. **105**(29): p. 9936-41.
8. Paeschke, K., J.A. Capra, and V.A. Zakian, *DNA replication through G-quadruplex motifs is promoted by the Saccharomyces cerevisiae Pif1 DNA helicase.* Cell, 2011. **145**(5): p. 678-91.
9. Kshirsagar, R., et al., *Probing the Potential Role of Non-B DNA Structures at Yeast Meiosis-Specific DNA Double-Strand Breaks.* Biophys J, 2017. **112**(10): p. 2056-2074.

10. Kendrick, S., et al., *The dynamic character of the BCL2 promoter i-motif provides a mechanism for modulation of gene expression by compounds that bind selectively to the alternative DNA hairpin structure.* J Am Chem Soc, 2014. **136**(11): p. 4161-71.
11. Miglietta, G., et al., *GC-elements controlling HRAS transcription form i-motif structures unfolded by heterogeneous ribonucleoprotein particle A1.* Sci Rep, 2015. **5**: p. 18097.
12. Kaiser, C.E., et al., *Insight into the Complexity of the i-Motif and G-Quadruplex DNA Structures Formed in the KRAS Promoter and Subsequent Drug-Induced Gene Repression.* J Am Chem Soc, 2017. **139**(25): p. 8522-8536.
13. Simonsson, T., M. Pribylova, and M. Vorlickova, *A nuclease hypersensitive element in the human c-myc promoter adopts several distinct i-tetraplex structures.* Biochem Biophys Res Commun, 2000. **278**(1): p. 158-66.
14. Guo, K., et al., *Intramolecularly folded G-quadruplex and i-motif structures in the proximal promoter of the vascular endothelial growth factor gene.* Nucleic Acids Res, 2008. **36**(14): p. 4598-608.
15. Bessler, J.B., J.Z. Torres, and V.A. Zakian, *The Pif1p subfamily of helicases: region-specific DNA helicases?* Trends Cell Biol, 2001. **11**(2): p. 60-5.
16. Ivessa, A.S., et al., *The Saccharomyces cerevisiae helicase Rrm3p facilitates replication past nonhistone protein-DNA complexes.* Mol Cell, 2003. **12**(6): p. 1525-36.
17. Ivessa, A.S., et al., *Saccharomyces Rrm3p, a 5' to 3' DNA helicase that promotes replication fork progression through telomeric and subtelomeric DNA.* Genes Dev, 2002. **16**(11): p. 1383-96.
18. Huber, M.D., D.C. Lee, and N. Maizels, *G4 DNA unwinding by BLM and Sgs1p: substrate specificity and substrate-specific inhibition.* Nucleic Acids Res, 2002. **30**(18): p. 3954-61.
19. Ghosh, A.K., et al., *RECQL4, the protein mutated in Rothmund-Thomson syndrome, functions in telomere maintenance.* J Biol Chem, 2012. **287**(1): p. 196-209.
20. Rogers, C.M., et al., *Yeast Hrq1 shares structural and functional homology with the disease-linked human RecQ4 helicase.* Nucleic Acids Res, 2017. **45**(9): p. 5217-5230.
21. Wu, Y., et al., *Biochemical characterization of Warsaw breakage syndrome helicase.* J Biol Chem, 2012. **287**(2): p. 1007-21.
22. Lerner, L.K., et al., *Timeless couples G-quadruplex detection with processing by DDX11 helicase during DNA replication.* EMBO J, 2020. **39**(18): p. e104185.

1st Revision - Editorial Decision 20th Sep 2023

Dr. Gideon Coster
Institute of Cancer Research
Cancer Biology
237 Fulham Road
London SW3 6JB
United Kingdom

20th Sep 2023

Re: EMBOJ-2023-114334R
Replication-induced DNA secondary structures drive fork uncoupling and breakage

Dear Gideon,

Thank you again for submitting your revised manuscript, as well as for your patience during it re-evaluation. We have now received feedback from all three original referees, who -I am pleased to say- are mostly satisfied with the revisions and improvements of the study. As you will see from the comments below, only referee 3 still has some specific questions, which I would invite you to incorporate and respond to in a final round of minor revision. The first point may require some statistical calculation and display, whereas the other points only require text changes and/or answers in the response letter.

Once we will have received this final version, we expect we should be able to swiftly proceed with formal acceptance and production of the manuscript!

With kind regards,

Hartmut

Hartmut Vodermaier, PhD
Senior Editor, The EMBO Journal
h.vodermaier@embojournal.org

*** PLEASE NOTE: All revised manuscript are subject to initial checks for completeness and adherence to our formatting guidelines. Revisions may be returned to the authors and delayed in their editorial re-evaluation if they fail to comply to the following requirements (see also our Guide to Authors for further information):

1) Every manuscript requires a Data Availability section (even if only stating that no deposited datasets are included). Primary datasets or computer code produced in the current study have to be deposited in appropriate public repositories prior to resubmission, and reviewer access details provided in case that public access is not yet allowed. Further information: embopress.org/page/journal/14602075/authorguide#dataavailability

2) Each figure legend must specify
- size of the scale bars that are mandatory for all micrograph panels
- the statistical test used to generate error bars and P-values
- the type error bars (e.g., S.E.M., S.D.)
- the number (n) and nature (biological or technical replicate) of independent experiments underlying each data point
- Figures may not include error bars for experiments with n<3; scatter plots showing individual data points should be used instead.

3) Revised manuscript text (including main tables, and figure legends for main and EV figures) has to be submitted as editable text file (e.g., .docx format). We encourage highlighting of changes (e.g., via text color) for the referees' reference.

4) Each main and each Expanded View (EV) figure should be uploaded as individual production-quality files (preferably in .eps, .tif, .jpg formats). For suggestions on figure preparation/layout, please refer to our Figure Preparation Guidelines: http://bit.ly/EMBOPressFigurePreparationGuideline

5) Point-by-point response letters should include the original referee comments in full together with your detailed responses to them (and to specific editor requests if applicable), and also be uploaded as editable (e.g., .docx) text files.

6) Please complete our Author Checklist, and make sure that information entered into the checklist is also reflected in the

manuscript; the checklist will be available to readers as part of the Review Process File. A download link is found at the top of our Guide to Authors: embopress.org/page/journal/14602075/authorguide

7) All authors listed as (co-)corresponding need to deposit, in their respective author profiles in our submission system, a unique ORCiD identifier linked to their name. Please see our Guide to Authors for detailed instructions.

8) Please note that supplementary information at EMBO Press has been superseded by the 'Expanded View' for inclusion of additional figures, tables, movies or datasets; with up to five EV Figures being typeset and directly accessible in the HTML version of the article. For details and guidance, please refer to:
embopress.org/page/journal/14602075/authorguide#expandedview

9) Digital image enhancement is acceptable practice, as long as it accurately represents the original data and conforms to community standards. If a figure has been subjected to significant electronic manipulation, this must be clearly noted in the figure legend and/or the 'Materials and Methods' section. The editors reserve the right to request original versions of figures and the original images that were used to assemble the figure. Finally, we generally encourage uploading of numerical as well as gel/blot image source data; for details see: embopress.org/page/journal/14602075/authorguide#sourcedata

At EMBO Press, we ask authors to provide source data for the main manuscript figures. Our source data coordinator will contact you to discuss which figure panels we would need source data for and will also provide you with helpful tips on how to upload and organize the files.

Further information is available in our Guide For Authors:
https://www.embopress.org/page/journal/14602075/authorguide

In the interest of ensuring the conceptual advance provided by the work, we recommend submitting a revision within 3 months (19th Dec 2023). Please discuss the revision progress ahead of this time with the editor if you require more time to complete the revisions. Use the link below to submit your revision:

Link Not Available

Referee #1:

In the revised manuscript the authors addressed many of the reviewers' comments and suggestions, which significantly improved it. I have two remaining concerns that the authors may want to address.

Major points:

(1) One of the major improvements is that the authors have now quantified the intensity of the normalized stall band based on the data from three independent experiments. It remains unclear to me, however, what differences between the original and mutant G4 motifs are significant and which ones are not (Figs. 1E and S2A), given that in many cases confident intervals overlap. Statistical significances shall be calculated and indicated on the figures.

(2) The authors' additional experiments with i-motif-mediated stalling revealed the lack of pH-dependence. As they acknowledge, this is very surprising given that the structure requires cytosine protonation. While some of those motifs fold into iM structures at neutral pH, they are much more stable at acidic pH, as is evident from the data in Tab. 2. Furthermore, the authors believe that there is some breakage of nascent DNA at iMs. Altogether, this is hard to comprehend. The only explanation that I can think of is that some protein component of their reconstituted replication system has high affinity to iM structures, which in turn promotes its latent endonuclease activity. Something for the authors to think about...

A minor point:

In the Introduction the authors state: "Examples of well-characterised secondary structures include hairpin (Gacy et al, 1995; Nadel Weisman-Shomer & Fry, 1995), G-quadruplex (G4) (Fry & Loeb, 1994) and intercalated-Motif (iM) (Gehring Leroy & Gueron, 1993) structures, which are thought to act as barriers to replication". The authors need to add triplex H-DNA, as it is well documented that this structure blocks DNA synthesis in vitro and, in some cases, in vivo (for recent comprehensive reviews see PMID: 32060097 and PMID: 36316397).

Referee #2:

The authors have satisfactorily addressed my concerns.

Referee #3:

I would like to congratulate the authors on the revised manuscript. They clarified in detail all my concerns

Replication-induced secondary structures drive fork uncoupling and breakage: reviewer comments

We thank the three referees for taking the time to review our updated manuscript after incorporating the feedback suggested during the first round of revision, which we feel significantly enhanced our manuscript. We are very pleased to have addressed the majority of comments from reviewers satisfactorily. We appreciate that there are still some minor points to be addressed, as highlighted by referee #1, which we outline our response to below.

Referee #1 (Remarks to author):

In the revised manuscript the authors addressed many of the reviewers' comments and suggestions, which significantly improved it. I have two remaining concerns that the authors may want to address.

Major points:

(1) One of the major improvements is that the authors have now quantified the intensity of the normalized stall band based on the data from three independent experiments. It remains unclear to me, however, what differences between the original and mutant G4 motifs are significant and which ones are not (Figs. 1E and S2A), given that in many cases confident intervals overlap. Statistical significances shall be calculated and indicated on the figures.

We thank the reviewer for highlighting the improvement in the manuscript by quantifying stalling intensities. We have, as suggested, calculated the statistical significances of stalling between wildtype versus mutated G4 and iM sequences using Mann-Whitney tests. The p values generated were as follows:

G4s:
Myc wt vs mut: 0.2
4xGGGT vs 3xGGGT: 0.1
CEB25 L111 (T) vs (A): 0.4

iMs (2x):
DUX wt vs mut: 0.2
SNORD wt vs mut: 0.1

Although these p-values suggest that the differences between stalling at wildtype versus mutated sequences is not statistically significant, we believe that this may be due to the fact that stalling at wildtype G4s or iMs is relatively weak. Despite this, the reduction in replication stalling we see upon mutation of these sequences is still consistent.

We have updated the figure legends to include these p-values and updated the text as follows:

Page 8 paragraph 1: "Consistent with our hypothesis, we saw a consistent, though not statistically significant, reduction in stalling with the mutated sequences (**Fig. 1D, 1E**)."

Page 8 paragraph 1: "When the iM-forming sequences DUX4L22 and SNORD112 were disrupted for their structure-forming ability (**Appendix Fig. S1B**), stalling was consistently reduced, albeit not statistically significantly so (**Fig. 1F, 1G**)."

(2) The authors' additional experiments with i-motif-mediated stalling revealed the lack of pH-dependence. As they acknowledge, this is very surprising given that the structure requires cytosine protonation. While some of those motifs fold into iM structures at neutral pH, they are much more stable at acidic pH, as is evident from the data in Tab. 2. Furthermore, the authors believe that there is some breakage of nascent DNA at iMs. Altogether, this is hard to comprehend. The only explanation that I can think of is that some protein component of their reconstituted replication system has high affinity to iM structures, which in turn promotes its latent endonuclease activity. Something for the authors to think about...

We thank the reviewer for pointing out these interesting results in our study. Whilst we were surprised that stalling at iMs was not exacerbated when we reduced the pH, we speculate that this may be due to the fact that all of the iMs tested in this study were selected for based on their ability to form structures at neutral pH. As a result, all of these iM sequences may form stable iM structures under the standard conditions of our replication assays at pH 7.6. It is possible that other iM structures which are less stable under physiological pH may induce stronger replisome arrest under more acidic pH conditions.

The question of what causes breakage of nascent DNA at iMs is intriguing and requires further experiments to decipher. One potential cause of this breakage is some contaminating nuclease activity in replication reactions, but this seems unlikely given that this phenomenon is only seen at iM-forming sequences and occurs in the same position each time. As the reviewer has suggested, another possibility is cleavage by a structure-specific endonuclease within the replisome components which directly targets iM structures. Further experiments are required to test each replisome component to determine if this is indeed the case. Another potential cause of breakage may be more physical, in that replication of iM structures somehow alters the DNA structure, leaving it more sensitive to breakage, potentially due to the exposure of ssDNA. This is consistent with the fact that inherent DNA breakage is more likely to occur at abasic sites [1]. This will be a focus of future studies.

A minor point:

In the Introduction the authors state: "Examples of well-characterised secondary structures include hairpin (Gacy et al, 1995; Nadel Weisman-Shomer & Fry, 1995), G-quadruplex (G4) (Fry & Loeb, 1994) and intercalated-Motif (iM) (Gehring Leroy & Gueron, 1993) structures, which are thought to act as barriers to replication". The authors need to add triplex H-DNA, as it is well documented that this structure blocks DNA synthesis in vitro and, in some cases, in vivo (for recent comprehensive reviews see PMID: 32060097 and PMID: 36316397).

We thank the reviewer for pointing out that we did not include reference to triplex structures. We have updated the manuscript accordingly:

Page 2 paragraph 2: "Examples of well-characterised secondary structures include hairpin [2, 3], triplex (H-DNA) [4], G-quadruplex (G4) [5] and intercalated-Motif (iM) [6] structures, which are thought to act as barriers to replication."

1. Taylor, M.R.G. and J.T.P. Yeeles, *Dynamics of Replication Fork Progression Following Helicase-Polymerase Uncoupling in Eukaryotes.* J Mol Biol, 2019. **431**(10): p. 2040-2049.
2. Gacy, A.M., et al., *Trinucleotide repeats that expand in human disease form hairpin structures in vitro.* Cell, 1995. **81**(4): p. 533-40.
3. Nadel, Y., P. Weisman-Shomer, and M. Fry, *The fragile X syndrome single strand d(CGG)n nucleotide repeats readily fold back to form unimolecular hairpin structures.* J Biol Chem, 1995. **270**(48): p. 28970-7.
4. Mirkin, S.M., et al., *DNA H form requires a homopurine-homopyrimidine mirror repeat.* Nature, 1987. **330**(6147): p. 495-7.
5. Fry, M. and L.A. Loeb, *The fragile X syndrome d(CGG)n nucleotide repeats form a stable tetrahelical structure.* Proc Natl Acad Sci U S A, 1994. **91**(11): p. 4950-4.
6. Gehring, K., J.L. Leroy, and M. Gueron, *A tetrameric DNA structure with protonated cytosine.cytosine base pairs.* Nature, 1993. **363**(6429): p. 561-5.

Dr. Gideon Coster
Institute of Cancer Research
Cancer Biology
237 Fulham Road
London SW3 6JB
United Kingdom

21st Sep 2023

Re: EMBOJ-2023-114334R1
Replication-induced DNA secondary structures drive fork uncoupling and breakage

Dear Dr. Coster,

Thank you for submitting your final revised manuscript for our consideration. I am pleased to inform you that we have now accepted it for publication in The EMBO Journal.

Your article will be processed for publication in The EMBO Journal by EMBO Press and Wiley, who will contact you with further information regarding production/publication procedures and license requirements. You will also be provided with page proofs after copy-editing and typesetting of main manuscript and expanded view figure files.

Should you be planning a Press Release on your article, please get in contact with embojournal@wiley.com as early as possible, in order to coordinate publication and release dates.

Thank you again for this contribution to The EMBO Journal and congratulations on a successful publication! Please consider us again in the future for your most exciting work.

Yours sincerely,

Hartmut Vodermaier

Hartmut Vodermaier, PhD
Senior Editor, The EMBO Journal
h.vodermaier@embojournal.org

EMBO Press Author Checklist

Corresponding Author Name: Gideon Coster
Journal Submitted to: EMBO Journal
Manuscript Number: EMBOJ-2023-114334

USEFUL LINKS FOR COMPLETING THIS FORM
The EMBO Journal - Author Guidelines
EMBO Reports - Author Guidelines
Molecular Systems Biology - Author Guidelines
EMBO Molecular Medicine - Author Guidelines

Reporting Checklist for Life Science Articles (updated January
This checklist is adapted from Materials Design Analysis Reporting (MDAR) Checklist for Authors. MDAR establishes a minimum set of requirements in transparent reporting in the life sciences (see Statement of Task: 10.31222/osf.io/9sm4x). Please follow the journal's guidelines in preparing your
Please note that a copy of this checklist will be published alongside your article.

Abridged guidelines for figures
1. Data
The data shown in figures should satisfy the following conditions:
- the data were obtained and processed according to the field's best practice and are presented to reflect the results of the experiments in an accurate and unbiased manner.
- ideally, figure panels should include only measurements that are directly comparable to each other and obtained with the same assay.
- plots include clearly labeled error bars for independent experiments and sample sizes. Unless justified, error bars should not be shown for technical
- if n<5, the individual data points from each experiment should be plotted. Any statistical test employed should be justified.
- Source Data should be included to report the data underlying figures according to the guidelines set out in the authorship guidelines on Data

2. Captions
Each figure caption should contain the following information, for each panel where they are relevant:
- a specification of the experimental system investigated (eg cell line, species name).
- the assay(s) and method(s) used to carry out the reported observations and measurements.
- an explicit mention of the biological and chemical entity(ies) that are being measured.
- an explicit mention of the biological and chemical entity(ies) that are altered/varied/perturbed in a controlled manner.
- the exact sample size (n) for each experimental group/condition, given as a number, not a range;
- a description of the sample collection allowing the reader to understand whether the samples represent technical or biological replicates (including how many animals, litters, cultures, etc.).
- a statement of how many times the experiment shown was independently replicated in the laboratory.
- definitions of statistical methods and measures:
  - common tests, such as t-test (please specify whether paired vs. unpaired), simple χ2 tests, Wilcoxon and Mann-Whitney tests, can be unambiguously identified by name only, but more complex techniques should be described in the methods section;
  - are tests one-sided or two-sided?
  - are there adjustments for multiple comparisons?
  - exact statistical test results, e.g., P values = x but not P values < x;
  - definition of 'center values' as median or average;
  - definition of error bars as s.d. or s.e.m.

Please complete ALL of the questions below.
Select "Not Applicable" only when the requested information is not relevant for your study.

Materials

Newly Created Materials	Information included in the manuscript?	In which section is the information available? (Reagents and Tools Table, Materials and Methods, Figures, Data Availability Section)
New materials and reagents need to be available; do any restrictions apply?	Yes	Materials and Methods

Antibodies	Information included in the manuscript?	In which section is the information available? (Reagents and Tools Table, Materials and Methods, Figures, Data Availability Section)
For **antibodies** provide the following information:<br>- Commercial antibodies: RRID (if possible) or supplier name, catalogue number and or/clone number<br>- Non-commercial: RRID or citation	Not Applicable	

DNA and RNA sequences	Information included in the manuscript?	In which section is the information available? (Reagents and Tools Table, Materials and Methods, Figures, Data Availability Section)
**Short novel DNA or RNA including primers, probes**: provide the sequences.	Yes	Materials and Methods, Tables

Cell materials	Information included in the manuscript?	In which section is the information available? (Reagents and Tools Table, Materials and Methods, Figures, Data Availability Section)
**Cell lines:** Provide species information, strain. Provide accession number in repository **OR** supplier name, catalog number, clone number, and/**OR** RRID.	Not Applicable	
**Primary cultures:** Provide species, strain, sex of origin, genetic modification status.	Not Applicable	
Report if the cell lines were recently **authenticated** (e.g., by STR profiling) and tested for mycoplasma contamination.	Not Applicable	

Experimental animals	Information included in the manuscript?	In which section is the information available? (Reagents and Tools Table, Materials and Methods, Figures, Data Availability Section)
**Laboratory animals or Model organisms:** Provide species, strain, sex, age, genetic modification status. Provide accession number in repository **OR** supplier name, catalog number, clone number, **OR** RRID.	Not Applicable	
**Animal observed in or captured from the field:** Provide species, sex, and age where possible.	Not Applicable	
Please detail **housing and husbandry conditions**.	Not Applicable	

Plants and microbes	Information included in the manuscript?	In which section is the information available? (Reagents and Tools Table, Materials and Methods, Figures, Data Availability Section)
**Plants:** provide species and strain, ecotype and cultivar where relevant, unique accession number if available, and source (including location for collected wild specimens).	Not Applicable	
**Microbes:** provide species and strain, unique accession number if available, and source.	Not Applicable	

Human research participants	Information included in the manuscript?	In which section is the information available? (Reagents and Tools Table, Materials and Methods, Figures, Data Availability Section)
If collected and within the bounds of privacy constraints report on age, sex and gender or ethnicity for all study participants.	Not Applicable	

Core facilities	Information included in the manuscript?	In which section is the information available? (Reagents and Tools Table, Materials and Methods, Figures, Data Availability Section)
If your work benefited from core facilities, was their service mentioned in the acknowledgments section?	Not Applicable	

Design

Study protocol	Information included in the manuscript?	In which section is the information available? (Reagents and Tools Table, Materials and Methods, Figures, Data Availability Section)
If study protocol has been **pre-registered, provide DOI in the manuscript**. For clinical trials, provide the trial registration number **OR** cite DOI.	Not Applicable	
Report the **clinical trial registration number** (at ClinicalTrials.gov or equivalent), where applicable.	Not Applicable	

Laboratory protocol	Information included in the manuscript?	In which section is the information available? (Reagents and Tools Table, Materials and Methods, Figures, Data Availability Section)
Provide DOI OR other citation details if **external detailed step-by-step protocols** are available.	Not Applicable	

Experimental study design and statistics	Information included in the manuscript?	In which section is the information available? (Reagents and Tools Table, Materials and Methods, Figures, Data Availability Section)
Include a statement about **sample size** estimate even if no statistical methods were used.	Not Applicable	
Were any steps taken to minimize the effects of subjective bias when allocating animals/samples to treatment (e.g. **randomization procedure**)? If yes, have they been described?	Not Applicable	
Include a statement about **blinding** even if no blinding was done.	Not Applicable	
Describe **inclusion/exclusion criteria** if samples or animals were excluded from the analysis. Were the criteria pre-established?<br><br>If sample or data points were omitted from analysis, report if this was due to attrition or intentional exclusion and provide justification.	Not Applicable	
For every figure, are **statistical tests** justified as appropriate? Do the data meet the assumptions of the tests (e.g., normal distribution)? Describe any methods used to assess it. Is there an estimate of variation within each group of data? Is the variance similar between the groups that are being statistically compared?	Not Applicable	

Sample definition and in-laboratory replication	Information included in the manuscript?	In which section is the information available? (Reagents and Tools Table, Materials and Methods, Figures, Data Availability Section)
In the figure legends: state number of times the experiment was **replicated** in laboratory.	Yes	In all figure legends as relevant
In the figure legends: define whether data describe **technical or biological replicates**.	Yes	All replicates are biological replicates

Ethics

Ethics	Information included in the manuscript?	In which section is the information available? (Reagents and Tools Table, Materials and Methods, Figures, Data Availability Section)
Studies involving **human participants**: State details of **authority granting ethics approval** (IRB or equivalent committee(s), provide reference number for approval.	Not Applicable	
Studies involving **human participants**: Include a statement confirming that **informed consent** was obtained from all subjects and that the experiments conformed to the principles set out in the WMA Declaration of Helsinki and the Department of Health and Human Services Belmont Report.	Not Applicable	
Studies involving **human participants:** For publication of **patient photos**, include a statement confirming that consent to publish was obtained.	Not Applicable	
Studies involving experimental **animals**: State details of **authority granting ethics approval** (IRB or equivalent committee(s), provide reference number for approval. Include a statement of compliance with ethical regulations.	Not Applicable	
Studies involving **specimen and field samples:** State if relevant **permits** obtained, provide details of authority approving study; if none were required, explain why.	Not Applicable	

Dual Use Research of Concern (DURC)	Information included in the manuscript?	In which section is the information available? (Reagents and Tools Table, Materials and Methods, Figures, Data Availability Section)
Could your study fall under dual use research restrictions? Please check biosecurity documents and list of **select agents and toxins** (CDC): https://www.selectagents.gov/sat/list.htm	Not Applicable	
If you used a select agent, is the security level of the lab appropriate and reported in the manuscript?	Not Applicable	
If a study is subject to dual use research of concern regulations, is the name of the **authority granting approval and reference number** for the regulatory approval provided in the manuscript?	Not Applicable	

Reporting

The MDAR framework recommends adoption of discipline-specific guidelines, established and endorsed through community initiatives. Journals have their own policy about requiring specific guidelines and recommendations to complement MDAR.

Adherence to community standards	Information included in the manuscript?	In which section is the information available? (Reagents and Tools Table, Materials and Methods, Figures, Data Availability Section)
State if relevant guidelines or checklists (e.g., **ICMJE, MIBBI, ARRIVE, PRISMA**) have been followed or provided.	Not Applicable	
For **tumor marker prognostic studies,** we recommend that you follow the **REMARK** reporting guidelines (see link list at top right). See author guidelines, under 'Reporting Guidelines'. Please confirm you have followed these guidelines.	Not Applicable	
For **phase II and III randomized controlled trials**, please refer to the **CONSORT** flow diagram (see link list at top right) and submit the CONSORT checklist (see link list at top right) with your submission. See author guidelines, under 'Reporting Guidelines'. Please confirm you have submitted this list.	Not Applicable	

Data Availability

Data availability	Information included in the manuscript?	In which section is the information available? (Reagents and Tools Table, Materials and Methods, Figures, Data Availability Section)
Have **primary datasets** been deposited according to the journal's guidelines (see 'Data Deposition' section) and the respective accession numbers provided in the Data Availability Section?	Not Applicable	
Were **human clinical and genomic datasets** deposited in a public access-controlled repository in accordance to ethical obligations to the patients and to the applicable consent agreement?	Not Applicable	
Are **computational models** that are central and integral to a study available without restrictions in a machine-readable form? Were the relevant accession numbers or links provided?	Not Applicable	
If publicly available data were reused, provide the respective **data citations in the reference list**.	Not Applicable	
